# Resignation in Working Women With Breast and Gynecologic Cancers

**DOI:** 10.1001/jamanetworkopen.2025.28844

**Published:** 2025-08-25

**Authors:** Masahiro Iwakura, Kengo Nagashima, Kisho Shimizu, Shinichi Tanihara, Kaori Terata, Teiichiro Yamazaki, Songee Jung, Takumi Kimura, Masakazu Terauchi, Kyoko Nomura

**Affiliations:** 1 Department of Environmental Health Science and Public Health, Akita University Graduate School of Medicine, Akita, Japan; 2 Biostatistics Unit, Clinical and Translational Research Centre, Keio University Hospital, Tokyo, Japan; 3 School of Medicine, Akita University Faculty of Medicine, Akita, Japan; 4 Department of Public Health, School of Medicine, Kurume University, Kurume-City, Fukuoka, Japan; 5 Department of Thoracic Surgery, Akita University Graduate School of Medicine, Akita, Japan; 6 Department of Medical Informatics, Akita University Graduate School of Medicine, Akita Japan; 7 Department of Women’s Health, Institute of Science Tokyo, Tokyo, Japan

## Abstract

**Importance:**

Diagnosis of breast and gynecologic cancers may hamper employment; little is known about who is at greater risk of resignation.

**Objective:**

To examine whether a breast or gynecologic cancer diagnosis is associated with resignation in working women and to identify high-risk subpopulations.

**Design, Setting, and Participants:**

This matched cohort study included women aged 15 to 58 years insured by the Japan Health Insurance Association. From April 2017 to March 2023, women newly diagnosed with breast, cervical, uterine, or ovarian cancer were identified and matched in a 1:10 ratio to working women without these cancers.

**Exposure:**

Cancer diagnosis was based on *International Statistical Classification of Diseases and Related Health Problems, Tenth Revision (ICD-10)*.

**Main Outcomes and Measures:**

The primary outcome was all-cause resignation, and the secondary outcome was a composite of resignation and death, both assessed during a 2-year follow-up. Stratified Cox proportional-hazards models were applied, with multiple imputation and covariate adjustments. Subgroup analyses by prespecified covariates assessed effect size moderation and interactions between exposure and these covariates.

**Results:**

The study included 99 964 women with cancer, including 59 452 women with breast cancer (median [IQR] age, 48 [44-53] years), 14 713 women with cervical cancer (median [IQR] age, 46 [39-51] years), 16 933 women with uterine cancer (median [IQR] age, 49 [44-53 years]), and 8866 women with ovarian cancer (median age [IQR] age, 47 [40-52] years), as well as 999 640 matched controls (594 520 for the breast cancer cohort, 147 130 for the cervical cancer cohort, 169 330 for the uterine cancer cohort, and 88 660 for the ovarian cancer cohort). Most women with cancer were working in the medical, health care, or welfare sector (32 123 women [32.1%]). During follow-up, resignation rates were significantly higher for women with cancer vs controls (breast cancer: 10 820 women [18.2%] vs 97 892 women [16.5%]; hazard ratio [HR], 1.18; 95% CI, 1.16-1.20; cervical cancer: 3296 women [22.4%] vs 27 476 women [18.7%]; HR, 1.31; 95% CI, 1.26-1.36; uterine cancer: 3161 women [18.7%] vs 27 786 women [16.4%]; HR, 1.24; 95% CI, 1.19-1.29; ovarian cancer: 2004 women [22.6%] vs 15 847 women [17.9%]; HR, 1.44; 95% CI, 1.37-1.51). Regarding the composite outcome of resignation and death, there was an increased risk for all cohorts, ranging from an HR of 1.25 (95% CI, 1.22-1.27) for breast cancer to 1.81 (95% CI, 1.73-1.89) for ovarian cancer. Among all 4 cancer cohorts, resignation risk was higher among women with older age, lower income, and a history of depression.

**Conclusions and Relevance:**

In this cohort study using a nationwide health claims database in Japan, breast and gynecologic cancers were associated with a higher resignation risk, especially among those with older age, lower income, and depression history. These findings suggest that developing targeted support interventions may benefit women at high resignation risk after a cancer diagnosis.

## Key Points

**Question:**

Is a diagnosis of breast or gynecologic cancer associated with job resignation among working women, and what factors are associated with higher risk?

**Findings:**

This cohort study of 99 964 working women in Japan younger than 58 years with a diagnosis of breast (59 452 women), cervical (14 713 women), uterine (16 933 women), and ovarian (8866 women) cancer and 999 640 matched controls found that a cancer diagnosis was associated with a higher risk of resignation, particularly among individuals with lower income, older age, and depression history.

**Meaning:**

These findings suggest that targeted workplace support may be necessary to help retain women with cancer in the workforce.

## Teaser

This cohort study analyzes associations of breast, uterine, cervical, and ovarian cancer with job resignation among working women in Japan.

## Introduction

Breast and gynecologic cancers commonly affect prime working-age women (ie, 30-59 years), unlike other major cancers (eg, lung or colorectal) that peak after 60 years of age.^1,2^ Furthermore, incidence of breast and uterine cancer is rising in the US and Japan.^1,2^ Therefore, these cancers substantially affect working women’s employment,^3,4,5^ even with improved survival rates.^6,7,8,9,10,11,12^ Employment disruption stems from multiple factors including symptoms and treatment burden,^3,4,13,14^ job characteristics,^13,14^ workplace accommodations and support,^13,14^ and personal circumstances such as marital status^15^ and having a partner who is employed.^16^ Additionally, the greater share of unpaid household and caregiving responsibilities that women often bear^17^ may also play a role.

Cancer survivors, particularly with breast or gynecologic cancer, face higher unemployment or altered employment compared with healthy controls.^5^ The economic burden of these cancers is also substantial, with indirect costs such as premature mortality and reduced productivity accounting for approximately 65%.^18,19,20^ Moreover, unemployment can cause financial hardship,^21,22^ reduced quality of life,^14,23^ treatment disruptions,^21,24^ and lower survival rates.^23,25^ Despite well-documented adverse consequences, critical knowledge gaps persist. Robust, population-based longitudinal data on resignation are scarcer for gynecologic cancers^3,4^ than for breast cancer.^5^ Moreover, precise identification of working women at higher risk of resignation, especially for diverse gynecologic cancers,^3,4^ remains poorly defined, hindering the development of targeted employment retention strategies.

To address these knowledge gaps, we used nationwide health insurance claims data from the Japan Health Insurance Association (JHIA). This study primarily aimed to quantify the risk of resignation following a breast or gynecologic cancer diagnosis compared with matched working women without these cancers. Additionally, exploratory analysis examined effect size moderation across various individual and disease-related factors to identify specific subpopulations of women at higher risk of resignation who may require dedicated job retention support.

## Methods

This matched cohort study utilized data from insured persons registered in the JHIA database. The institutional review board of Akita University approved this study on May 26, 2023, waiving informed consent because it used retrospective, anonymized data. This study followed the Strengthening the Reporting of Observational Studies in Epidemiology (STROBE) reporting guideline.^26^ Key terms and definitions are presented in Table 1, and the design diagram is shown in eFigure 1 in Supplement 1.

**Table 1.** Definitions of the Terms in This Study

Term	Definitions
Observation period	The observation period in this study was from the beginning of fiscal year 2015 to the end of fiscal year 2022 (April 1, 2015, to March 31, 2023).
Cohort entry day	The month and year when an employee enrolled in the Japan Health Insurance Association health insurance system.
Exposure	Initial diagnosis of breast (cohort 1), cervical (cohort 2), uterine (cohort 3), or ovarian (cohort 4) cancer.
Nonexposure	No diagnosis of the 4 cancers during the observation period.
Index date	
Exposure group	The month of the initial diagnosis of each cancer.
Nonexposure group	The same month of the corresponding index date of the women with cancer.
Lookback period	From 24 mo before the index date to the index date.
Outcome	
Primary	Resignation from the index date to the end of the observation period or 24 mo after the index date.
Secondary	Resignation or death from the index date to the end of the observation period or 24 mo after the index date.
Event day	The month of resignation (or death) after the index date.

### Data Sources

Japan’s health insurance system consists of 3 types: employee health insurance, national health insurance (for self-employed and unemployed individuals), and the medical care system for seniors (aged 75 years and older). The JHIA is the largest employee health insurer in Japan. The JHIA database contains administrative records, insurance claims (recorded monthly), and annual health examination results for approximately 40 million employees (mainly at small to midsized companies^27^) and their dependents. In fiscal year 2022, these individuals represented 31.7% of Japan’s population.^28^ Among these, approximately 9.6 million working-age women were registered as employees (approximately 26% of Japan’s working-age women).^28,29^ The observational period was April 1, 2015, to March 31, 2023.

### Population and Exposure Definition

The study population comprised insured women newly diagnosed with breast (cohort 1), cervical (cohort 2), uterine (cohort 3), or ovarian (cohort 4) cancer between April 1, 2017, and March 31, 2023. Cancer incidence was defined as having an initial definitive diagnosis based on the *International Statistical Classification of Diseases and Related Health Problems, Tenth Revision (ICD-10)*. The cancer diagnosis date served as the index date for the women with cancer.

To minimize prevalent user bias,^30^ women needed at least 24 months of JHIA health insurance registration before the index date (the lookback period)^31^ and no prior definitive diagnosis or surgical record of the corresponding cancer. We also excluded women with 1 of the 6 Health Insurance Claims Review and Reimbursement Services^32^ modifier codes for recurrence because they were unlikely to newly develop cancer (eTable 1 in Supplement 1). Additionally, to accurately measure resignation due to cancer onset, we included women aged 15 to 58 years because approximately 75% of Japanese small- to middle-sized companies have mandatory retirement at 60 years.^33,34^ The cancer detection algorithms and code lists are detailed in eAppendix 1 and eTables 2 to 4 in Supplement 1, with the code lists developed with a breast surgeon (K.T.) and an obstetrician and gynecologist (M.T.).

### Nonexposure Definition and Matching Process

Matching candidates were insured women with no definitive diagnosis of the 4 cancers during the observational period. Ten candidates were then randomly assigned per woman (1:10 ratio) with cancer as matched individuals. Matching was based on year of birth, month and year of cohort entry day, and age at index date (±2 years).

### Outcome Measure and Handling of Censoring

Outcomes and censoring were determined using JHIA qualification loss date and reasons. eAppendix 2 in Supplement 1 details the definition of resignation and death via JHIA qualification loss, as well as their specific operationalization for this study. The primary outcome was time from index date to all-cause resignation within the 2-year follow-up.^35,36^ Women with cancer and matched individuals were followed from the index date until (1) resignation, (2) death, (3) health insurance qualification loss for other reasons (eg, overseas assignment or unpaid insurance premiums), (4) the end of the observational period (March 31, 2023), or (5) 24 months from index date, whichever occurred earliest. The secondary outcome was a composite of resignation and death, representing workforce attrition.

### Potential Covariates

Baseline covariates collected from the JHIA database included demographics, health examination results, and past and current medical histories associated with the exposure and outcome^37^ (eFigure 1 in Supplement 1). Demographics included age, residency region, affiliated entity classification, industrial classifications (per Health Insurance Act of Japan),^38^ monthly income, and workplace years of service. Latest annual health examination^39^ results (2-year lookback) provided body mass index, alcohol consumption frequency, smoking status, and physical activity levels. Previous health examination experience was considered good health behavior.^40^ Past or current medical histories included depression,^41^ alcohol or drug abuse,^42^ dementia,^42,43^ and other cancers.^42,43^ eAppendix 3 in Supplement 1 details covariate definitions and measurements.

### Statistical Analysis

All statistical analyses utilized R version 4.2 (R Project for Statistical Computing). Missing covariates were addressed by multiple imputation, generating 300 imputed datasets with the mice package; estimates were combined using the Rubin rules.^44^ eAppendix 4 in Supplement 1 details methods and assumptions for handling missing data. Statistical significance for testing interactions between cancer incidence and prespecified subgroups was defined as a 2-sided *P* < .05.

Grambsch-Therneau score tests and scaled Schoenfeld residual plots^45^ assessed the proportional hazard assumption. Time-to-event analyses used cause-specific Cox proportional hazards models stratified on matched pairs to estimate the hazard ratios (HRs) and 95% CIs. All models included the specified covariates, excluding histories of substance abuse (alcohol or drug), dementia, and other cancers.

Potential effect size moderation was examined in 13 prespecified subgroups by including an interaction term in the statistical model: age, residency region, monthly income, years of service, affiliated entity classification, industrial classifications, health examination history, body mass index, alcohol drinking frequency, smoking status, engagement in moderate to vigorous physical activity (defined as ≥2 moderate-intensity sessions per week, ≥30 minutes per session, sustained ≥1 year), engagement in light physical activity (defined as walking or comparable activity ≥60 minutes daily), and history of depression. Interactions between cancer incidence and subgroups were determined using stratified Cox proportional hazards models. All subgroup analyses used imputed datasets, with adjustments on the same covariates.

Finally, 2 prespecified sensitivity analyses were conducted. First, we fitted crude Cox proportional-hazards models using imputed datasets for the primary and secondary outcomes. Second, the Fine-Gray model^46^ was fitted, treating death as a competing risk with covariate adjustment (primary outcome only). During peer review, an additional sensitivity analysis—a subgroup analysis of the primary outcome by the fiscal year of index date—assessed the potential impact of the COVID-19 pandemic.

## Results

### Participant Selection and Baseline Characteristics

This study included 99 964 women diagnosed with cancer (April 2017 to March 2023), including 59 452 women with breast cancer (median [IQR] age, 48 [44-53] years), 14 713 women with cervical cancer (median [IQR] age, 46 [39-51] years), 16 933 women with uterine cancer (median [IQR] age, 49 [44-53 years]), and 8866 women with ovarian cancer (median [IQR] age, 47 [40-52] years). Each participant was matched with 10 individuals without cancer, resulting in a total of 999 640 controls (594 520 for the breast cancer cohort, 147 130 for the cervical cancer cohort, 169 330 for the uterine cancer cohort, and 88 660 for the ovarian cancer cohort). Table 2 presents participant selection, and Table 3 details baseline characteristics of the participants. Among the 4 cohorts, 27 654 (27.7%) resided in the Kanto region and 17 656 (17.7%) resided in Chubu. Median (IQR) monthly income ranged from ¥220 000 (¥180 000-¥280 000) to ¥240 000 (¥180 000-¥300 000), and median (IQR) job tenure ranged from 6 (4-11) years to 7 (4-13) years. The most frequent industry classification was medical, health care, and welfare (32 123 women [32.1%]), followed by wholesale and retail trade (14 347 women [14.4%]), and manufacturing (13 208 women [13.2%]). Depression history was more common in women with cancer (4699 women [4.7%]) than in matched individuals (6055 individuals [0.6%]) across cohorts.

**Table 2.** Selection Process of Women With Cancer and Matched Individuals

Selection process criteria	Individuals by cohort, No.			
	Cohort 1: breast cancer	Cohort 2: cervical cancer	Cohort 3: uterine cancer	Cohort 4: ovarian cancer
Women with cancer^a^				
Women with a definitive cancer diagnosis	275 883	69 694	57 228	45 353
Diagnosed received between April 1, 2017, and March 31, 2023	204 061	50 848	45 327	34 440
Age <58 y at the index date	171 776	46 860	38 961	30 249
Lookback period ≥2 y	60 614	17 803	19 631	13 400
Without operation during the lookback period	59 452	NA	NA	8866
Without operation and *ICD-10* code C55 during the lookback period	NA	14 713	16 933	NA
Matched individuals^b^	594 520	147 130	169 330	88 660

Abbreviations: *ICD-10*, *International Statistical Classification of Diseases and Related Health Problems, Tenth Revision*; NA, not applicable.

^a^ From 25 082 935 insured women in the database during the observational period.

^b^ From a total of 24 078 513 candidates.

**Table 3.** Baseline Characteristics of Women With Breast or Gynecologic Cancer and Matched Individuals

Characteristic	Participants, No. (%)^a^							
	Cohort 1		Cohort 2		Cohort 3		Cohort 4	
	Matched individuals (n = 594 520)	Breast cancer (n = 59 452)	Matched individuals (n = 147 130)	Cervical cancer (n = 14 713)	Matched individuals (n = 169 330)	Uterine cancer (n = 16 933)	Matched individuals (n = 88 660)	Ovarian cancer (n = 8866)
Age, median (IQR), y	48 (44-53)	48 (44-53)	45 (39-51)	46 (39-51)	49 (44-53)	49 (44-53)	47 (40-52)	47 (40-52)
Residency region								
Hokkaido	25 255 (4.2)	2760 (4.6)	6209 (4.2)	605 (4.1)	7224 (4.3)	746 (4.4)	3734 (4.2)	274 (3.1)
Tohoku	50 743 (8.5)	4740 (8.0)	12 620 (8.6)	1210 (8.2)	14 485 (8.6)	1272 (7.5)	7423 (8.4)	712 (8.0)
Kanto	157 960 (26.6)	16 566 (27.9)	39 805 (27.1)	3665 (24.9)	44 525 (26.3)	4885 (28.8)	23 530 (26.5)	2538 (28.6)
Chubu	115 834 (19.5)	11 096 (18.7)	28 127 (19.1)	2217 (15.1)	33 006 (19.5)	2761 (16.3)	17 330 (19.5)	1582 (17.8)
Kinki	96 468 (16.2)	10 163 (17.1)	24 078 (16.4)	2258 (15.3)	27 556 (16.3)	2499 (14.8)	14 513 (16.4)	1494 (16.9)
Chugoku and Shikoku	66 448 (11.2)	6040 (10.2)	16 046 (10.9)	1525 (10.3)	18 836 (11.1)	1925 (11.4)	9786 (11.0)	1052 (11.9)
Kyushu and Okinawa	81 812 (13.8)	8087 (13.6)	20 245 (13.8)	3233 (22.0)	23 698 (14.0)	2845 (16.8)	12 344 (13.9)	1214 (13.7)
Monthly income, median (IQR), 1000 ¥^b^^,^^c^	220 (180-300)	240 (180-300)	220 (180-280)	220 (180-280)	220 (180-300)	220 (180-300)	220 (180-280)	240 (180-300)
Missing, No.	229	0	45	0	52	0	25	0
Years of service^b^								
Median (IQR)	7 (4-13)	7 (4-13)	6 (3-11)	6 (4-11)	7 (4-13)	7 (4-13)	7 (4-12)	7 (4-12)
Missing, No.	229	0	45	0	52	0	25	0
Job classification corporate	579 444 (97.5)	57 885 (97.3)	143 317 (97.4)	14 324 (97.4)	165 118 (97.5)	16 469 (97.3)	86 390 (97.4)	8634 (97.4)
Industry classifications								
Construction	26 772 (4.5)	2960 (5.0)	6246 (4.2)	723 (4.9)	7705 (4.6)	773 (4.6)	3823 (4.3)	364 (4.1)
Manufacturing	81 791 (13.8)	7798 (13.1)	19 710 (13.4)	1884 (12.8)	23 378 (13.8)	2312 (13.7)	12 062 (13.6)	1214 (13.7)
Information and communications	7610 (1.3)	985 (1.7)	2098 (1.4)	192 (1.3)	2218 (1.3)	240 (1.4)	1195 (1.3)	155 (1.7)
Transport and postal services	17 271 (2.9)	1688 (2.8)	4077 (2.8)	448 (3.0)	4819 (2.8)	489 (2.9)	2534 (2.9)	278 (3.1)
Wholesale and retail trade	81 434 (13.7)	8524 (14.3)	20 257 (13.8)	2092 (14.2)	23 337 (13.8)	2411 (14.2)	12 260 (13.8)	1320 (14.9)
Real estate and goods, rental, and leasing	13 182 (2.2)	1581 (2.7)	3328 (2.3)	374 (2.5)	3638 (2.1)	372 (2.2)	1993 (2.2)	222 (2.5)
Scientific research, professional, and technical services	24 094 (4.1)	2925 (4.9)	6240 (4.2)	552 (3.8)	6781 (4.0)	727 (4.3)	3719 (4.2)	418 (4.7)
Accommodations and eating and drinking services	17 170 (2.9)	1498 (2.5)	4281 (2.9)	481 (3.3)	4946 (2.9)	406 (2.4)	2621 (3.0)	235 (2.7)
Living-related, personal, and amusement service	17 776 (3.0)	1681 (2.8)	4979 (3.4)	620 (4.2)	5090 (3.0)	457 (2.7)	2863 (3.2)	311 (3.5)
Education and learning support	11 869 (2.0)	1276 (2.1)	3204 (2.2)	296 (2.0)	3338 (2.0)	324 (1.9)	1830 (2.1)	189 (2.1)
Medical, health care, and welfare	194 909 (32.8)	18 754 (31.5)	48 419 (32.9)	4978 (33.8)	55 755 (32.9)	5559 (32.8)	29 195 (32.9)	2832 (31.9)
Compound services	8215 (1.4)	816 (1.4)	1855 (1.3)	206 (1.4)	2348 (1.4)	288 (1.7)	1112 (1.3)	118 (1.3)
Services	56 701 (9.5)	5490 (9.2)	13 841 (9.4)	1225 (8.3)	15 824 (9.3)	1526 (9.0)	8337 (9.4)	748 (8.4)
Government^d^	25 233 (4.2)	2383 (4.0)	6050 (4.1)	407 (2.8)	7156 (4.2)	717 (4.2)	3514 (4.0)	323 (3.6)
Other^e^	10 493 (1.8)	1093 (1.8)	2545 (1.7)	235 (1.6)	2997 (1.8)	332 (2.0)	1602 (1.8)	139 (1.6)
Health examination history: yes	371 453 (62.5)	40 167 (67.5)	80 600 (54.7)	8241 (56.0)	103 825 (61.3)	10 707 (63.2)	50 550 (57.0)	5258 (59.3)
Body mass index								
Median (IQR)^f^	21.6 (19.6-24.4)	21.5 (19.6-24.2)	21.4 (19.4-24.2)	21.2 (19.2-23.9)	21.6 (19.5-24.4)	22.5 (20.0-26.7)	21.5 (19.4-24.3)	21.7 (19.4-24.8)
Missing, No.	223 067	19 285	66 530	6472	65 505	6226	38 110	3608
Alcohol drinking frequency, No./total No. (%)								
Hardly or never	169 279/321 173 (52.7)	17 710/34 947 (50.6)	35 447/67 500 (52.5)	3333/6879 (48.5)	46 766/90 165 (51.9)	5031/9205 (54.7)	22 626/42 670 (53.0)	2448/4414 (55.5)
Sometimes	97 666/321 173 (30.4)	10 802/34 947 (30.9)	21 006/67 500 (31.1)	2189/6879 (31.8)	27 129/90 165 (30.1)	2816/9205 (30.6)	12 954/42 670 (30.4)	1307/4414 (29.6)
Every day	54 228/321 173 (16.9)	6435/34 947 (18.4)	11 047/67 500 (16.4)	1357/6879 (19.7)	15 270/90 165 (16.9)	1358/9205 (14.8)	7090/42 670 (16.6)	659/4414 (14.9)
Missing, No.	273 347	24 505	79 630	7834	80 165	7728	45 990	4452
Smoking status, No./total No. (%)								
Yes	62 277/367 177 (17.0)	6314/39 662 (15.9)	13 432/77 403 (17.4)	1853/7830 (23.7)	17 143/102 038 (16.8)	1518/10 543 (14.4)	8362/48 788 (17.1)	791/5042 (15.7)
Missing, No.	227 343	19 790	69 727	6883	67 292	6390	39 872	3824
Engagement in MVPA								
Yes, No./total No. (%)	45 141/317 905 (14.2)	4881/34 649 (14.2)	9345/66 770 (14.0)	934/6809 (13.7)	12 577/88 170 (14.3)	1314/9117 (14.4)	5882/42 155 (14.0)	619/4372 (14.2)
Missing, No.	276 615	24 803	80 360	7904	81 160	7816	46 505	4494
Engagement in LPA								
Yes, No./total No. (%)	109 699/318 061 (34.5)	11 635/34 666 (33.6)	23 177/66 783 (34.7)	2401/6819 (35.2)	30 407/88 182 (34.5)	3076/9106 (33.8)	14 617/42 176 (34.7)	1500/4362 (34.4)
Missing, No.	276 459	24 786	80 347	7894	81 148	7827	46 484	4504
History of depression	3694 (0.6)	2684 (4.5)	803 (0.5)	733 (5.0)	1064 (0.6)	874 (5.2)	494 (0.6)	408 (4.6)
History of alcohol drug abuse	<10	<10	<10	<10	<10	<10	<10	<10
History of dementia	0	0	0	0	0	0	0	0
History of other cancer	20 (<0.1)	22 (<0.1)	<10	12 (<0.1)	<10	13 (<0.1)	<10	11 (0.1)

Abbreviations: LPA, light physical activity; MVPA, moderate to vigorous physical activity.

^a^ To ensure anonymization, we presented the data as less than 10 when the frequency of each cell was below this threshold.

^b^ Regarding monthly income and years of service, data were complete for all cancer cases, whereas some missing values were present in the matched control individuals. An investigation into the missingness did not reveal any clear cause, leading us to assume these variables were missing completely at random. We assumed the other covariates were missing at random.

^c^ To convert Japanese yen to US dollars, multiply by 0.0067.

^d^ Except where classified.

^e^ Combination of agriculture, forestry, and fisheries; mining and quarrying of stone and gravel; electricity, gas, heat supply, and water; and finance and insurance.

^f^ Calculated as weight in kilograms divided by height in meters squared.

### Resignation and Death During the 2-Year Follow-Up Period

During the follow-up, resignation rates for women with cancer vs matched individuals were 10 820 women (18.2%) vs 97 892 women (16.5%) for breast cancer, 3296 women (22.4%) vs 27 476 women (18.7%) for cervical cancer, 3161 women (18.7%) vs 27 786 women (16.4%) for uterine cancer, and 2004 women (22.6%) vs 15 847 women (17.9%) for ovarian cancer. The Figure presents cumulative resignation incidence curves. Death rates for women with cancer vs matched individuals were 684 women (1.2%) vs 407 women (0.1%) for breast cancer, 429 women (2.9%) vs 83 women (0.1%) for cervical cancer, 350 women (2.1%) vs 112 women (0.1%) for uterine cancer, and 508 women (5.7%) vs 66 women (0.1%) for ovarian cancer. Consequently, rates of the composite outcome (resignation and death) were higher in women with cancer than in the matched individuals (breast cancer: 11 504 women [19.4%] vs 98 299 women [16.5%]; cervical cancer: 3725 women [25.3%] vs 27 559 women [18.7%]; uterine cancer: 3511 women [20.7%] vs 27 898 women [16.5%]; ovarian cancer: 2512 women [28.3%] vs 15 913 women [17.9%]) (eFigure 2 in Supplement 1).

**Figure.**
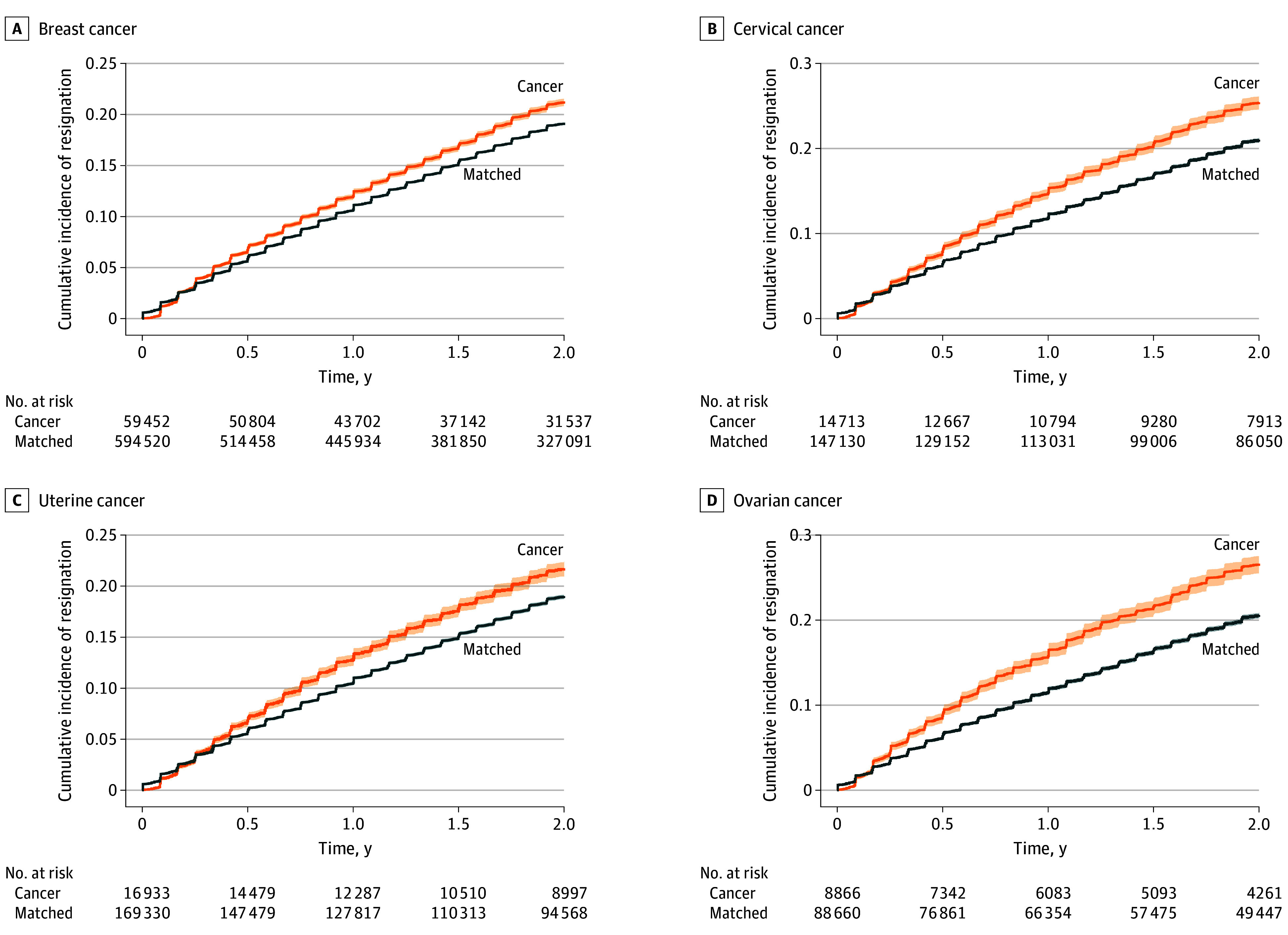
Cumulative Incidence Curves of Resignation After Initial Cancer Diagnosis Each panel presents the cumulative incidence curve of resignation for women with breast cancer (A), cervical cancer (B), uterine cancer (C), and ovarian cancer (D) and matched individuals. The x-axis is the time (years) from the index date (women with cancer, the month of the initial cancer diagnosis; matched individuals, the index date of the corresponding patients), while the y-axis is the cumulative probability of resignation or death. The orange line represents women with cancer, the blue line represents matched individuals, and the shaded area represents the 95% CIs. The number at risk for each time interval is presented under each x-axis.

### Association of Initial Cancer Diagnosis With Outcomes

The proportional-hazards assumption was not seriously violated in all cohorts (eFigures 3 and 4 in Supplement 1). Cox regression analysis indicated that women with cancer were more likely to resign than matched individuals in all cohorts (Table 4). The risk was highest for ovarian cancer (HR, 1.44; 95% CI, 1.37-1.51), followed by cervical (HR, 1.31; 95% CI, 1.26-1.36), uterine (HR, 1.24; 95% CI, 1.19-1.29), and breast (HR, 1.18; 95% CI, 1.16-1.20) cancer. Similarly, the HR for resignation and death was also higher in women with cancer across cohorts (Table 4), again with ovarian cancer having the highest risk (HR, 1.81; 95% CI, 1.73-1.89), followed by cervical cancer (HR, 1.48; 95% CI, 1.43-1.53), uterine cancer (HR, 1.37; 95% CI, 1.32-1.42), and breast cancer (HR, 1.25; 95% CI, 1.22-1.27).

**Table 4.** Association of Breast and Gynecologic Cancer Incidence With Resignation^a^

Outcomes	Risk of resignation, HR (95% CI)			
	Cohort 1: breast cancer	Cohort 2: cervical cancer	Cohort 3: uterine cancer	Cohort 4: ovarian cancer
Primary analysis				
Resignation (adjusted)^b^	1.18 (1.16-1.20)	1.31 (1.26-1.36)	1.24 (1.19-1.29)	1.44 (1.37-1.51)
Resignation or death (adjusted)^b^	1.25 (1.22-1.27)	1.48 (1.43-1.53)	1.37 (1.32-1.42)	1.81 (1.73-1.89)
Sensitivity analysis				
Resignation	1.12 (1.10-1.14)	1.24 (1.19-1.28)	1.17 (1.19-1.22)	1.35 (1.29-1.42)
Resignation (adjusted subdistribution)^b^^,^^c^	1.17 (1.15-1.20)	1.29 (1.24-1.34)	1.22 (1.18-1.27)	1.40 (1.34-1.47)
Resignation or death	1.19 (1.16-1.21)	1.40 (1.35-1.44)	1.30 (1.25-1.34)	1.70 (1.63-1.77)

Abbreviation: HR, hazard ratio.

^a^ All the models used matched individuals as references. The results of using multiple imputation for missing covariates are presented here.

^b^ Adjusted for age, residency region, classification of the affiliated entity, industrial classifications, monthly income, year of service, health checkup history, body mass index, alcohol drinking frequency, smoking status, physical activity (engagement in moderate to vigorous physical activity and engagement in light physical activity), and history of depression.

^c^ Competing risk analysis was conducted using the Fine and Gray model to estimate subdistribution hazard ratios, treating death as a competing risk.

### Potential Effect Size Moderation of the Participants’ Characteristics

Resignation risk varied slightly by cancer type and contextual factors. In cervical cancer, regional variation was significant (*P *for interaction = .04). In breast cancer, risk differed by industry (*P *for interaction < .001) and was highest in the government sector. Lack of health examination history was also associated with higher risk (lack of health examination history: HR, 1.21; 95% CI, 1.17-1.25; health examination history: HR, 1.15; 95% CI, 1.12-1.18; *P *for interaction = .02). Common factors for associated with increased risk of resignation across all cancer cohorts included older age (<median vs ≥median), lower income (<median vs ≥median), and depression history. Longer job tenure (<median vs ≥median) was associated with increased risk of resignation for all cohorts except for breast cancer. Women with cancer with depression history had a nearly 2-fold increase in risk of resignation compared with those without in all cohorts. Details are shown in eFigures 5-8 in Supplement 1.

### Results of the Sensitivity Analysis

While HRs and 95% CIs showed slight deviations, the results of the 2 prespecified sensitivity analyses were consistent with the primary analysis (Table 4). The subgroup analysis by the fiscal year of index date, conducted as an additional sensitivity analysis, indicated that resignation rates and HRs had slight deviations but maintained overall consistent trends across fiscal years for all cohorts (eTable 5 in Supplement 1).

## Discussion

This matched cohort study found that women with breast or gynecologic cancer were more likely to resign than women without cancer (matched controls).^5,13,14,47^ Subgroup analysis indicated that working women with relatively older age, lower income, or a depression history faced a higher risk of resignation. Potential heterogeneity was observed in job tenure, residency regions, and industry classifications. Furthermore, sensitivity analysis suggested that this association of cancer diagnosis with resignation remained consistent throughout the observation period, including during the COVID-19 pandemic.

Resignation risk after initial cancer diagnosis varies by location^3,5,36^; in this study, it was highest for ovarian cancer, followed by cervical, uterine, and breast cancer. Differences in cancer stage at diagnosis may partly explain this variation. For example, ovarian cancer is often diagnosed at a more advanced stage than cervical, uterine, or breast cancer.^48,49,50^ Previous research also found that later-stage cancer was associated with a higher resignation risk than earlier-stage cancer.^13,51,52,53^ Hence, ovarian cancer diagnosis resulted in a higher resignation risk than other cancers. This study also found associations of all 4 cancer diagnoses and resignation, with an 18% to 44% increased resignation risk for insured women compared with matched individuals. When using a composite outcome (resignation and death), such risk was 1.25 to 1.81 times higher than that of matched individuals. This finding underscores the substantial contribution of these cancers to workforce attrition and associated productivity loss.

Beyond confirming established factors associated with resignation, such as older age,^13,14,23,54^ lower income,^13,14,23,54^ residency region,^14,48,55^ and depression history,^14,23,56^ subgroup analyses revealed specific vulnerabilities among Japanese working women. The higher prevalence of depression history among patients with cancer (approximately 5%; 8-10 times higher than in matched individuals) is consistent with increased depression prevalence after cancer diagnosis but before treatment^57^ and throughout the disease course.^58,59^ Furthermore, the large-magnitude association with a 2-fold higher resignation risk, even after covariate adjustments, highlighted the urgency of mental health interventions. Conversely, prior evidence on how industry classifications,^54^ job tenure,^13,51^ and lifestyle behaviors^13^ impact employment varied among cancer types. In this study, longer job tenure was associated with higher resignation risk across cervical, uterine, and ovarian cohorts, even after age adjustment. This pattern may relate to Japan’s retirement allowance system—where lump-sum payments, unlike in many Western countries, increase with tenure and are often given for voluntary resignations—potentially easing resignation for employees with health issues. The interaction between cancer diagnosis and industry classification was statistically significant only for breast cancer, suggesting potential interindustry heterogeneity. For instance, among 15 industrial sectors, higher resignation risks (HR ≥1.18—the HR point estimate for breast cancer) were observed in sectors like government, manufacturing, wholesales, medical and welfare or services, possibly due to inflexible work hours, nonregular employment, or physically demanding tasks, suggesting that sector-specific challenges warrant consideration. Notably, women with breast cancer who underwent regular health examinations were less likely to resign; this may be because breast cancer screening, often an optional component of general health examinations, leads to higher screening rates and earlier detection, which in turn may support continued employment as earlier-stage cancer is associated with a lower resignation risk.^13,51,52,53^ These findings provide a clearer identification of at-risk individuals and possible reasons, paving the way for more targeted support.

The specific vulnerabilities identified in this study emphasize the need for tailored and proactive support systems. Given the higher prevalence and substantial impact of depression among cancer patients,^57,58,59^ regular mental health examinations and workplace-based services—such as mental health counseling and stress management programs—may support continued employment for women with cancer. For those with longer tenure for whom resignation may appear financially viable due to retirement allowances, financial counseling exploring alternatives to resignation, alongside workplace accommodations,^55^ could improve retention. Industry-specific challenges require addressing; in physically demanding or inflexible sectors, promoting adaptable work arrangements (eg, modified duties, telework, and expanded leave systems especially for nonregular employees) and assisting employers with their implementation is critical. While broad strategies for prevention,^60,61^ early detection,^60,61^ and awareness remain essential, our findings underscore the importance of more nuanced interventions. Tailoring support to women’s mental health, financial circumstances (including tenure-based benefits), and industry demands may enhance work-treatment balance and job continuity for women with breast and gynecologic cancers in Japan. Despite national differences in systems like retirement benefits, these insights can help inform global strategies to support working women with these cancers.

### Strengths and Limitations

This study has 4 key strengths. First, using a nationwide administrative database reduced opportunistic sampling bias. Second, analyses were performed separately for each cancer type because cancer location impacts the association of diagnosis with resignation. We found differences in HRs between cancer types. Third, we matched or adjusted for various potential covariates (eg, birth year, age, and history of depression), leading to less biased estimations. Fourth, to the best of our knowledge, this is the largest cohort study investigating the association of breast or gynecologic cancer diagnosis with resignation among working women. These findings indicate that the association observed in working women in other countries^3,5,14^ also holds true for those in Japan.

This study included several limitations. First, residual confounding may have affected our results due to lack of information on several factors. Specifically, we lacked data on cancer severity, household status, and the specific types and intensity of cancer treatments (eg, extent of surgery, chemotherapy regimens, and radiotherapy use). Because treatment modalities can significantly influence employment outcomes, as suggested by previous research,^3,4,13^ our inability to adjust for these treatment-related factors represents a source of unmeasured confounding. While we focused on insured women who worked at least 20 hours per week during the lookback period, potentially reducing some baseline heterogeneity, future research should incorporate measures of disease severity, details regarding socioeconomic status, and comprehensive information on cancer treatments to better control for such potential confounders. Second, our investigation period was from 2015 to 2023, which may be a limited period for identifying the incidence of cancer. However, we employed a 2-year lookback period to exclude prevalent cases.^31^ Additionally, we excluded women with 1 of 6 Health Insurance Claims Review and Reimbursement Services modifier codes related to recurrence, indicating that those with such codes were most likely nonincident cases.^31^ Third, we assessed past and current medical histories solely based on associated *ICD-10* codes. Because this method could result in information bias, we used validated disease definitions^41,42,43^ and excluded suspected cases. Fourth, we were unable to distinguish between different reasons for resignation (eg, cancer, other diseases, relocation, and mandatory retirement). To address this, we shortened follow-up and restricted the index date age to 58 years. Layoffs and terminations are uncommon in Japan, compared with Western countries, due to labor laws and cultural factors that prioritize job security. Hence, we believe resignations for other reasons were minimized. Fifth, we only enrolled insured women to measure resignation because information on dependent’s resignation was unavailable. Such selection may have limited the generalizability of our results to dependents, who generally have lower incomes than insured women in Japan.

## Conclusions

In this matched cohort study using a nationwide health claims database in Japan, women with breast or gynecologic cancer faced a higher risk of resignation, especially those with older age, lower income, and depression history. These findings highlight the need for targeted workplace support to help retain women with cancer in the workforce. In addition to encouraging cancer screening, such support may include regular mental health screening for early detection and intervention, as well as financial counseling when needed, both of which appear beneficial in promoting work continuity by improving the balance between work and treatment.

## Notes

Supplement 1. **eFigure 1.** Study Design Diagrams for Investigating the Association Between Breast and Gynecologic Cancer Onset and Resignation **eTable 1.** List of Modifier Codes for Exclusion **eAppendix 1.** Details for Each Cancer Detection Algorithm **eTable 2.** List of Diagnostic Codes for the Inclusion of Each Cancer Type **eTable 3.** List of Diagnostic Codes for the Exclusion of Each Cancer Type **eTable 4.** List of Surgical Codes for the Exclusion of Each Cancer Type **eAppendix 2.** Definition and Ascertainment of Outcomes (Resignation and Death) using the JHIA Database **eAppendix 3.** Details of the Covariate Definitions and Measurements **eAppendix 4.** Details of Missing Data Handling **eFigure 2.** Cumulative Incidence Curves of Composite Outcome (Resignation or Death) After the Initial Cancer Diagnosis **eFigure 3.** Scaled Schoenfeld Residual Plots for Testing the Proportional-Hazards Assumption of the Primary Outcome **eFigure 4.** Scaled Schoenfeld Residual Plots for Testing the Proportional-Hazards Assumption of the Secondary Outcome (Resignation or Death) **eFigure 5.** Subgroup Analyses for the Associations Between Initial Diagnosis of Breast Cancer and Resignation in 13 Prespecified Covariates **eFigure 6.** Subgroup Analyses for the Associations Between Initial Diagnosis of Cervical Cancer and Resignation in 13 Prespecified Covariates **eFigure 7.** Subgroup Analyses for the Associations Between Initial Diagnosis of Uterine Cancer and Resignation in 13 Prespecified Covariates **eFigure 8.** Subgroup Analyses for the Associations Between Initial Diagnosis of Ovarian Cancer and Resignation in 13 Prespecified Covariates **eTable 5.** Results of an Additional Sensitivity Analysis Stratified by Fiscal Year of Index Date to Assess the Potential Impact of the COVID-19 Pandemic on the Association Between Initial Diagnosis of Cancers and Resignation

Supplement 2.
Data Sharing Statement
