# Supplementary material for: Resignation in Working Women With Breast and Gynecologic Cancers
Source: JAMA Netw Open. 2025 Aug 25;8(8):e2528844. doi: 10.1001/jamanetworkopen.2025.28844 (PMC12379106; doi:10.1001/jamanetworkopen.2025.28844)
Supplement: Supplement 1. — eFigure 1. Study Design Diagrams for Investigating the Association Between Breast and Gynecologic Cancer Onset and Resignation eTable 1. List of Modifier Codes for Exclusion eAppendix 1. Details for Each Cancer Detection Algorithm eTable 2. List of Diagnostic Codes for the Inclusion of Each Cancer Type eTable 3. List of Diagnostic Codes for the Exclusion of Each Cancer Type eTable 4. List of Surgical Codes for the Exclusion of Each Cancer Type eAppendix 2. Definition and Ascertainment of Outcomes (Resignation and Death) using the JHIA Database eAppendix 3. Details of the Covariate Definitions and Measurements eAppendix 4. Details of Missing Data Handling eFigure 2. Cumulative Incidence Curves of Composite Outcome (Resignation or Death) After the Initial Cancer Diagnosis eFigure 3. Scaled Schoenfeld Residual Plots for Testing the Proportional-Hazards Assumption of the Primary Outcome eFigure 4. Scaled Schoenfeld Residual Plots for Testing the Proportional-Hazards Assumption of the Secondary Outcome (Resignation or Death) eFigure 5. Subgroup Analyses for the Associations Between Initial Diagnosis of Breast Cancer and Resignation in 13 Prespecified Covariates eFigure 6. Subgroup Analyses for the Associations Between Initial Diagnosis of Cervical Cancer and Resignation in 13 Prespecified Covariates eFigure 7. Subgroup Analyses for the Associations Between Initial Diagnosis of Uterine Cancer and Resignation in 13 Prespecified Covariates eFigure 8. Subgroup Analyses for the Associations Between Initial Diagnosis of Ovarian Cancer and Resignation in 13 Prespecified Covariates eTable 5. Results of an Additional Sensitivity Analysis Stratified by Fiscal Year of Index Date to Assess the Potential Impact of the COVID-19 Pandemic on the Association Between Initial Diagnosis of Cancers and Resignation [file jamanetwopen-e2528844-s001.pdf]

Supplemental Online Content

Iwakura M, Nagashima K, Shimizu K, et al. Resignation in working women with breast and gynecologic cancers. JAMA Network Open. 2025;8(8): e2528844. doi:10.1001/jamanetworkopen.2025.28844

eFigure 1. Study Design Diagrams for Investigating the Association Between Breast and Gynecologic Cancer Onset and Resignation
eTable 1. List of Modifier Codes for Exclusion
eAppendix 1. Details for Each Cancer Detection Algorithm
eTable 2. List of Diagnostic Codes for the Inclusion of Each Cancer Type
eTable 3. List of Diagnostic Codes for the Exclusion of Each Cancer Type
eTable 4. List of Surgical Codes for the Exclusion of Each Cancer Type
eAppendix 2. Definition and Ascertainment of Outcomes (Resignation and Death) using the JHIA Database
eAppendix 3. Details of the Covariate Definitions and Measurements
eAppendix 4. Details of Missing Data Handling
eFigure 2. Cumulative Incidence Curves of Composite Outcome (Resignation or Death) After the Initial Cancer Diagnosis
eFigure 3. Scaled Schoenfeld Residual Plots for Testing the Proportional-Hazards Assumption of the Primary Outcome
eFigure 4. Scaled Schoenfeld Residual Plots for Testing the Proportional-Hazards Assumption of the Secondary Outcome (Resignation or Death)
eFigure 5. Subgroup Analyses for the Associations Between Initial Diagnosis of Breast Cancer and Resignation in 13 Prespecified Covariates
eFigure 6. Subgroup analyses for the Associations Between Initial Diagnosis of Cervical Cancer and Resignation in 13 Prespecified Covariates
eFigure 7. Subgroup analyses for the Associations Between Initial Diagnosis of Uterine Cancer and Resignation in 13 Prespecified Covariates
eFigure 8. Subgroup analyses for the Associations Between Initial Diagnosis of Ovarian Cancer and Resignation in 13 Prespecified Covariates
eTable 5. Results of an Additional Sensitivity Analysis Stratified by Fiscal Year of Index Date to Assess the Potential Impact of the COVID-19 Pandemic on the Association between Initial Diagnosis of Cancers and Resignation

This supplemental material has been provided by the authors to give readers additional information about their work.

© 2025 Iwakura M et al. *JAMA Network Open*.

eFigure 1. Study Design Diagrams for Investigating the Association Between Breast and Gynecologic Cancer Onset and Resignation

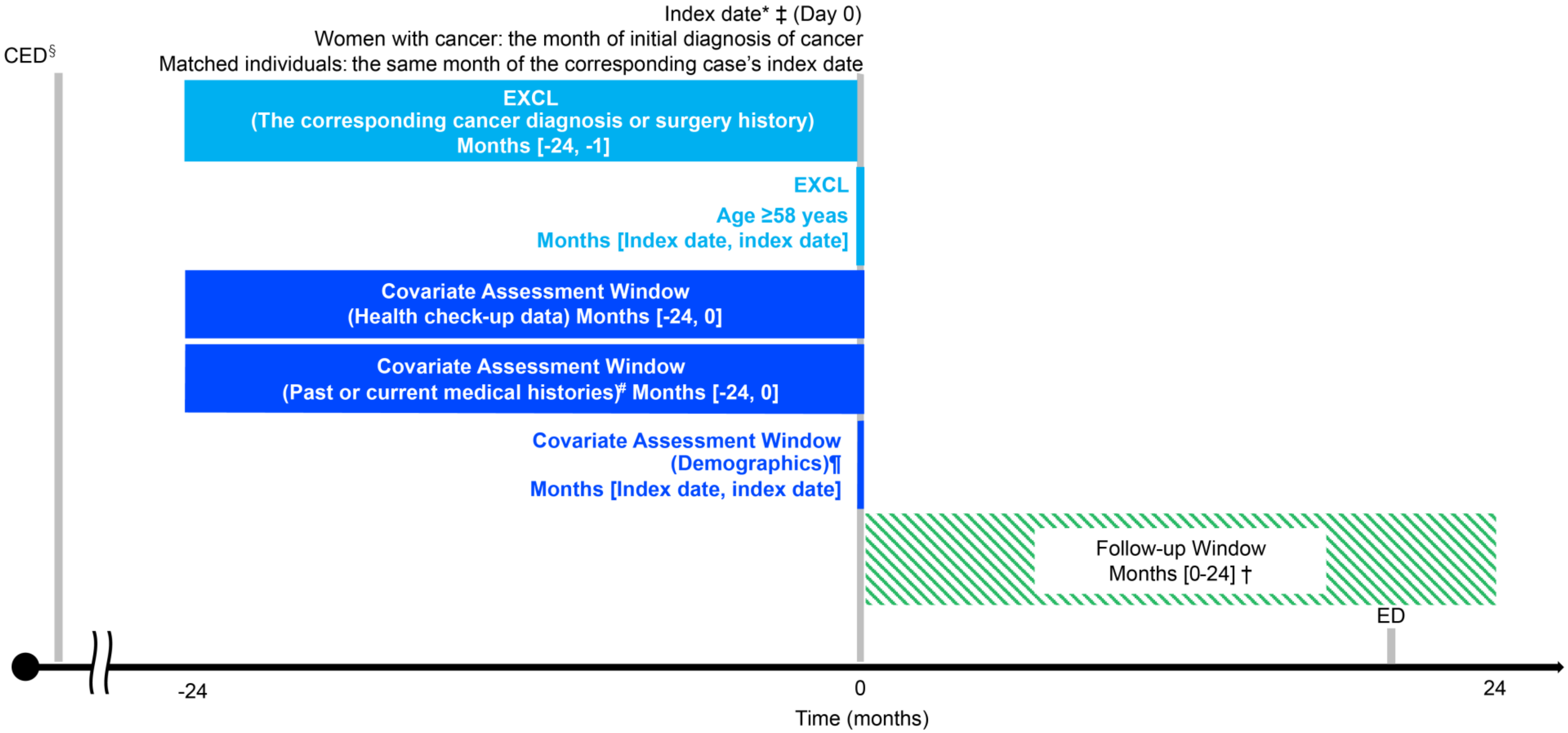

The observation period of this study was from the beginning of the fiscal year 2015 to the end of the fiscal year 2022 (April 1, 2015, to March 31, 2023).

* Index date was defined as the month when a participant received an initial diagnosis of breast (cohort 1), cervical (cohort 2), uterine (cohort 3), or ovarian (cohort 4) cancer in a large nationwide database from the Japan Health Insurance Association. The index date of the matched individuals was the same date as the corresponding woman with cancer.

§ CED was defined as the month when an employee enrolled in the health insurance system of the Japan Health Insurance Association.

© 2025 Iwakura M et al. *JAMA Network Open*.

$ The covariates retrieved from the health checkup results included body mass index, alcohol drinking frequency, smoking status, and engagement in physical activity.

Past of current medical histories included depression, alcohol or drug abuse, dementia, and cancers except for breast, cervical, uterine, and ovarian cancers.

Demographics included age, residency region, monthly income, years of service at the current workplace, classification of the affiliated entity, and industrial classification at the index date.

† Censored at the first incident of resignation, death, loss of health insurance qualification for reasons other than resignation or death, 24-month, or end of study period.

‡ Matched individuals were matched on the basis of the year of birth, the year and month of CED, and age at the index date.

Abbreviations: CED, cohort entry day; ED, event day; EXCL, Exclusion Assessment Window.

© 2025 Iwakura M et al. *JAMA Network Open*.

eTable 1. List of Modifier Codes for Exclusion

HICRRS Modifier Codes	Names in English
4024	Recurrence
4025	Recurrent
8016	Postoperative recurrence
4031	Post-resection recurrence
8063	Relapse of
8065	Recurrence of

Abbreviations: HICRRS Illness Codes, illness codes of the Health Insurance Claims Review & Reimbursement Services.

© 2025 Iwakura M et al. *JAMA Network Open*.

eAppendix 1. Details for Each Cancer Detection Algorithm

Breast Cancer: Cohort 1

The incidence of breast cancer was defined as having an initial and definitive diagnosis of breast cancer (*ICD-10* code C50) after a two-year lookback period (see eTable 2 in Supplement 1). We excluded women with the following HICRRS Illness Codes:[1] breast cancer recurrence (code 1749009); locally recurrent breast cancer (code 8849815); and chest wall recurrence after breast cancer surgery (code 8849816). We also excluded women with a diagnosis code (*ICD-10* code C50) or HICRRS surgical code[39] during the lookback period (see eTables 3–4 in Supplement 1).

Cervical Cancer: Cohort 2

We defined cervical cancer incidence as having an initial and definitive diagnosis of cervical cancer *(ICD-10* code C53) after a two-year lookback period (see eTable 2 in Supplement 1). We excluded women with *ICD-10* code C55 or HICRRS illness code 8834242 (stump, cervical), due to the uncertainty of the cancer location. We also excluded women with a diagnostic code (*ICD-10* code C53, except for HICRRS illness code 8834242 or C55) or HICRRS surgical code during the lookback period (see eTables 3–4 in Supplement 1).

Uterine Cancer: Cohort 3

We identified uterine cancer incidence as having an initial and definitive diagnosis of uterine body cancer (*ICD-10* code C54) after a two-year lookback period (see eTable 2 in Supplement 1). We excluded women with *ICD-10* code C55, HICRRS codes 1799005 (uterine sarcoma), 8846283 (uterine carcinosarcoma), or 1820003 (recurrence of uterine cancer) because we could not detect cancer location or incidence cases. We also excluded women with a diagnostic code (*ICD-10* codes C54 or C55) or HICRRS surgical code during the lookback period (see eTables 3–4 in Supplement 1).

Ovarian Cancer: Cohort 4

The incidence of ovarian cancer was defined as having an initial and definitive diagnosis of breast cancer (*ICD-10* code C56) after a two-year lookback period (see eTable 2 in Supplement 1). We excluded women with HICRRS code 8848712 (recurrence of ovarian cancer) because we assumed that those with this code are non-incidence cases. We also excluded women with a diagnostic code (*ICD-10* code C56) or HICRRS surgical code during the lookback period (see eTables 3–4 in Supplement 1).

Supplemental References

1. Health Insurance Claims Review & Reimbursement Services. Name of the Illness Master (Last updated May 2024). Accessed September 9, 2024.

eTable 2. List of Diagnostic Codes for the Inclusion of Each Cancer Type

Cancer types	ICD-10 Codes	HICRRS Illness Codes	Names in English
Breast	C500	8838489	Paget's disease
Breast	C500	8845450	Breast cancer of the nipple region
Breast	C500	8845452	Breast cancer of the areolar
Breast	C501	8838483	Central breast cancer
Breast	C502	8838476	Upper inner quadrant breast cancer
Breast	C503	8838465	Lower inner quadrant breast cancer
Breast	C504	8838475	Upper outer quadrant breast cancer
Breast	C505	8838464	Lower outer quadrant breast cancer
Breast	C506	8848843	Axillary breast cancer
Breast	C508	8845451	Peripheral breast cancer
Breast	C508	8848690	Ectopic breast cancer
Breast	C509	1749008	Breast cancer
Breast	C509	1749011	Malignant tumor of the mammary gland
Breast	C509	1749015	Malignant breast tumor
Breast	C509	8830917	Inflammatory breast cancer
Breast	C509	8842759	Advanced breast cancer
Breast	C509	8848722	Invasive carcinoma of no special type
Breast	C509	8848743	Multiple breast cancer
Breast	C509	8848773	Tubule forming type
Breast	C509	8849183	Scirrhous type
Breast	C509	8849184	Solid type
Breast	C509	8849699	HER2 positive breast cancer
Breast	C509	8851374	HER2-low breast cancer
Breast	C509	8851421	Invasive lobular carcinoma
Breast	C509	1749004	Postoperative breast cancer
Breast	C509	1749017	Breast sarcoma
Breast	C509	8845025	Liposarcoma of the breast
Breast	C509	8848647	Fibrosarcoma of the breast
Breast	C509	8842665	Malignant phyllodes tumor
Breast	C509	8848646	Angiosarcoma of the breast
Cervical	C530	1800003	Carcinoma (cancer) of the cervical canal
Cervical	C531	8834243	Carcinoma (cancer) of the vaginal portion of cervix
Cervical	C538	8848883	Carcinoma (cancer) of the squamocolumnar junction of the cervix uteri

© 2025 Iwakura M et al. *JAMA Network Open*.

Cervical	C539	1809004	Cervical (cervix) cancer (carcinoma)<br>Carcinoma (cancer) of the cervix uteri
Cervical	C539	8844722	Adenocarcinoma of the cervix uteri
Cervical	C539	8842739	Microinvasive carcinoma of the cervix uteri
Uterine	C540	8847757	Carcinoma (cancer) of the isthmus of uterus
Uterine	C541	1820005	Endometrial cancer (carcinoma)
Uterine	C541	8848717	Endometrioid carcinoma of the uterine body
Uterine	C541	1799007	Endometrial stromal sarcoma
Uterine	C542	1799006	Uterine leiomyosarcoma
Uterine	C543	8847758	Carcinoma of the fundus uteri
Uterine	C549	1820002	Endometrial cancer (carcinoma)<br>Carcinoma (cancer) of the uterine body (of the corpus uteri)
Uterine	C549	8848716	Cancer of the uterine body (of the corpus uteri)
Ovarian	C56	1830005	[Epithelial] ovarian cancer
Ovarian	C56	1830008	Ovarian sarcoma
Ovarian	C56	8846984	Ovarian carcinoid
Ovarian	C56	8846347	Ovarian carcinosarcoma
Ovarian	C56	1830014	Ovarian choriocarcinoma
Ovarian	C56	1830027	Ovarian embryonal carcinoma
Ovarian	C56	1830003	Ovarian cancer
Ovarian	C56	8848796	Ovarian small cell carcinoma
Ovarian	C56	8848800	Ovarian immature teratoma
Ovarian	C56	8849005	Ovarian mucinous [cyst] adenocarcinoma<br>Mucinous [cyst] adenocarcinoma of the ovary
Ovarian	C56	8847437	Ovarian germ cell tumors
Ovarian	C56	8848799	Ovarian squamous cell carcinoma
Ovarian	C56	8848801	Ovarian clear cell adenocarcinoma
Ovarian	C56	8847438	Ovarian yolk sac tumor<br>Ovarian primitive endodermal tumor
Ovarian	C56	8848802	Ovarian endometrioid adenocarcinoma
Ovarian	C56	8848795	Ovarian serous [cyst] adenocarcinoma<br>Serous [cyst] adenocarcinoma of the ovary
Ovarian	C56	1830009	Ovarian dysgerminoma
Ovarian	C56	8848765	Poorly differentiated ovarian Sertoli–Leydig cell tumor

Abbreviations: ICD-10 Codes, the International Statistical Classification of Diseases, 10th Revision; HICRRS Illness Codes,

© 2025 Iwakura M et al. *JAMA Network Open*.

illness codes of the Health Insurance Claims Review & Reimbursement Services.

We referred to General Rules for Clinical and Pathological Recording of Breast Cancer, 18th Edition published by the Japanese Breast Cancer Society for the Japanese notation of breast cancer and Obstetrics and Gynecology Glossary, 4th revised edition published by The Japan Society of Obstetrics and Gynecology for the Japanese notation of gynecological cancers.

A breast surgeon (KT) and an obstetrician and gynecologist (MT) translated for those not included in these books.

© 2025 Iwakura M et al. *JAMA Network Open*.

eTable 3. List of Diagnostic Codes for the Exclusion of Each Cancer Type

Cancer types	ICD-10 Codes	HICRRS Illness Codes	Names in English
Breast	C500	20072604	Paget's disease
Breast	C500	20093766	Breast cancer of the nipple region
Breast	C500	20093767	Breast cancer of the areolar
Breast	C501	20079567	Central breast cancer
Breast	C502	20079568	Upper inner quadrant breast cancer
Breast	C503	20079569	Lower inner quadrant breast cancer
Breast	C504	20079570	Upper outer quadrant breast cancer
Breast	C505	20079571	Lower outer quadrant breast cancer
Breast	C506	20101696	Axillary breast cancer
Breast	C508	20093765	Peripheral breast cancer
Breast	C508	20101549	Ectopic breast cancer
Breast	C509	20054921	Inflammatory breast cancer
Breast	C509	20064279	Postoperative breast cancer
Breast	C509	20072471	Breast cancer
Breast	C509	20072473	Recurrent breast cancer
Breast	C509	20072530	Malignant tumor of the mammary gland
Breast	C509	20072606	Malignant breast tumor
Breast	C509	20072647	Breast sarcoma
Breast	C509	20087445	Malignant phyllodes tumor
Breast	C509	20087536	Advanced breast cancer
Breast	C509	20092871	Liposarcoma of the breast
Breast	C509	20101331	Angiosarcoma of the breast
Breast	C509	20101332	Fibrosarcoma of the breast
Breast	C509	20101550	Multiple breast cancer
Breast	C509	20101552	Invasive carcinoma of no special type
Breast	C509	20101553	Tubule forming type
Breast	C509	20102233	Solid type
Breast	C509	20102235	Scirrhous type
Breast	C509	20103570	Chest wall recurrence of breast cancer
Breast	C509	20103572	Local recurrence of breast cancer
Breast	C509	20103582	HER2 positive breast cancer
Breast	C509	20107343	HER2-low breast cancer
Breast	C509	20107345	Invasive lobular carcinoma
Cervical	C530	1800003	Carcinoma (cancer) of the cervical canal

© 2025 Iwakura M et al. *JAMA Network Open*.

Cervical	C531	8834243	Carcinoma (cancer) of the vaginal portion of cervix
Cervical	C538	8848883	Carcinoma (cancer) of the squamocolumnar junction of the cervix uteri
Cervical	C539	1809004	Cervical (cervix) cancer (carcinoma)<br>Carcinoma (cancer) of the cervix uteri
Cervical	C539	8844722	Adenocarcinoma of the cervix uteri
Cervical	C539	8842739	Microinvasive carcinoma of the cervix uteri
Cervical	C55	1799003	Carcinoma (cancer) uteri
Cervical	C55	1799004	Recurrence of Carcinoma (cancer) uteri
Uterine	C540	8847757	Carcinoma (cancer) of the isthmus of uterus
Uterine	C541	1820005	Endometrial cancer (carcinoma)
Uterine	C541	8848717	Endometrioid carcinoma of the uterine body
Uterine	C541	1799007	Endometrial stromal sarcoma
Uterine	C542	1799006	Uterine leiomyosarcoma
Uterine	C543	8847758	Carcinoma of the fundus uteri
Uterine	C549	1820002	Endometrial cancer (carcinoma)<br>Carcinoma (cancer) of the uterine body (of the corpus uteri)
Uterine	C549	8848716	Cancer of the uterine body (of the corpus uteri)
Uterine	C549	1820003	Recurrence of endometrial cancer (carcinoma)
Uterine	C55	1799003	Carcinoma (cancer) uteri
Uterine	C55	1799004	Recurrence of Carcinoma (cancer) uteri
Ovarian	C56	1830005	[Epithelial] ovarian cancer
Ovarian	C56	1830008	Ovarian sarcoma
Ovarian	C56	8846984	Ovarian carcinoid
Ovarian	C56	8846347	Ovarian carcinosarcoma
Ovarian	C56	1830014	Ovarian choriocarcinoma
Ovarian	C56	1830027	Ovarian embryonal carcinoma
Ovarian	C56	1830003	Ovarian cancer
Ovarian	C56	8848796	Ovarian small cell carcinoma
Ovarian	C56	8848800	Ovarian immature teratoma
Ovarian	C56	8849005	Ovarian mucinous [cyst] adenocarcinoma<br>Mucinous [cyst] adenocarcinoma of the ovary
Ovarian	C56	8847437	Ovarian germ cell tumors
Ovarian	C56	8848799	Ovarian squamous cell carcinoma
Ovarian	C56	8848801	Ovarian clear cell adenocarcinoma
Ovarian	C56	8847438	Ovarian yolk sac tumor

© 2025 Iwakura M et al. *JAMA Network Open*.

			Ovarian primitive endodermal tumor
Ovarian	C56	8848802	Ovarian endometrioid adenocarcinoma
Ovarian	C56	8848795	Ovarian serous [cyst] adenocarcinoma<br>Serous [cyst] adenocarcinoma of the ovary
Ovarian	C56	1830009	Ovarian dysgerminoma
Ovarian	C56	8848765	Poorly differentiated ovarian Sertoli–Leydig cell tumor
Ovarian	C56	8849004	Mature cystic teratoma of the ovary, malignant transformation

Abbreviations: ICD-10 Codes, the International Statistical Classification of Diseases, 10th Revision; HICRRS Illness Codes, illness codes of the Health Insurance Claims Review & Reimbursement Services.

We referred to General Rules for Clinical and Pathological Recording of Breast Cancer, 18th Edition published by the Japanese Breast Cancer Society for the Japanese notation of breast cancer and Obstetrics and Gynecology Glossary, 4th revised edition published by The Japan Society of Obstetrics and Gynecology for the Japanese notation of gynecological cancers.

A breast surgeon (KT) and an obstetrician and gynecologist (MT) translated for those not included in these books.

© 2025 Iwakura M et al. *JAMA Network Open*.

eTable 4. List of Surgical Codes for the Exclusion of Each Cancer Type

Cancer types	HICRRS Surgical Codes	Names in English
Breast	150121610	Surgery for malignant breast tumor (simple mastectomy)
Breast	150121710	Surgery for malignant breast tumor (mastectomy without pectoral muscle resection))
Breast	150121810	Surgery for malignant breast tumor (mastectomy with pectoral muscle resection)
Breast	150121910	Surgery for malignant breast tumor (extended mastectomy with axillary lymph node dissection)
Breast	150122150	Surgery for malignant breast tumor and bilateral axillary lymph node dissection
Breast	150316510	Surgery for malignant breast tumor (mastectomy without axillary lymph node dissection)
Breast	150386410	Surgery for malignant breast tumor (nipple sparing mastectomy without axillary lymph node dissection)
Breast	150386510	Surgery for malignant breast tumor (nipple sparing mastectomy with axillary lymph node dissection)
Breast	150121410	Mastectomy
Breast	150413710	Mastectomy (hereditary breast and ovarian cancer syndrome patient)
Cervical	150216510	Amputation of the cervix uteri, cervical amputation
Cervical	150281950	trachelectomy
Cervical	150216650	Laser ablation of cervical intraepithelial neoplasia or carcinoma in situ
Cervical	150327010	Laser ablation of cervical intraepithelial neoplasia or carcinoma in situ
Cervical	150281750	Photodynamic therapy of cervical intraepithelial neoplasia or early-stage cervical cancer
Cervical	150327110	Photodynamic therapy of cervical intraepithelial neoplasia or early-stage cervical cancer
Cervical	150217410	Supravaginal (supracervical) amputation of the uterus<br>Subtotal (supravaginal) hysterectomy
Cervical	150366010	Laparoscopic supravaginal (supracervical) amputation of the uterus<br>Laparoscopic subtotal (supravaginal) hysterectomy
Cervical	150217510	Total hysterectomy

© 2025 Iwakura M et al. *JAMA Network Open*.

Cervical	150272250	Total laparoscopic hysterectomy
Cervical	150217610	Excision of tumor of the broad ligament of the uterus
Cervical	150327210	Laparoscopic excision of tumor of the broad ligament of the uterus
Cervical	150217710	Surgery of cancer uteri
Cervical	150409810	Laparoscopic surgery of cervical cancer
Uterine	150217510	Total hysterectomy
Uterine	150272250	Total laparoscopic hysterectomy
Uterine	150217610	Excision of tumor of the broad ligament of the uterus
Uterine	150327210	Laparoscopic excision of tumor of the broad ligament of the uterus
Uterine	150217710	Surgery of cancer uteri
Uterine	150379810	Laparoscopic surgery of endometrial cancer
Uterine	150409310	Robotic-assisted laparoscopic surgery of endometrial cancer
Ovarian	150219710	Partial resection of the ovary (open)
Ovarian	150264710	Partial resection of the ovary (laparoscopic)
Ovarian	150220010	Excision of ovarian/adnexal tumor (open)
Ovarian	150270010	Excision of ovarian/adnexal tumor (laparoscopic)
Ovarian	150220150	Salpingectomy (open)
Ovarian	150268050	Salpingectomy (laparoscopic)
Ovarian	150220250	Excision of salpingeal tumor (open)
Ovarian	150268150	Excision of salpingeal tumor (laparoscopic)
Ovarian	150220450	Surgery of hematosalpinx (open)
Ovarian	150268250	Surgery of hematosalpinx (laparoscopic)
Ovarian	150220710	Surgery of ovarian/adnexal cancer (carcinoma)

Abbreviations: ICD-10 Codes, the International Statistical Classification of Diseases, 10th Revision; HICRRS Illness Codes, illness codes of the Health Insurance Claims Review & Reimbursement Services.

We referred to General Rules for Clinical and Pathological Recording of Breast Cancer, 18th Edition published by the Japanese Breast Cancer Society for the Japanese notation of breast cancer and Obstetrics and Gynecology Glossary, 4th revised edition published by The Japan Society of Obstetrics and Gynecology for the Japanese notation of gynecological cancers.

A breast surgeon (KT) and an obstetrician and gynecologist (MT) translated for those not included in these books.

© 2025 Iwakura M et al. *JAMA Network Open*.

eAppendix 2. Definition and Ascertainment of Outcomes (Resignation and Death) using the JHIA Database

In Japan's employee health insurance system, employees are generally unable to choose their specific health insurer. If a company is covered by the Japan Health Insurance Association (JHIA), its employees are mandatorily enrolled in the JHIA system concurrently with their hiring. Consequently, cessation of employment with that company results in the simultaneous loss of JHIA insured status. This linkage allows the period of JHIA insurance coverage to serve as a proxy for continuous employment at the same company.

The JHIA database provides information on the dates of acquiring and losing insurance qualification, as well as the specific reason for the loss of qualification. This study leveraged this information to define outcomes as follows:

- Resignation (Primary Outcome): An individual was considered to have resigned if the reason for loss of JHIA qualification was documented as "resignation" (退職). The date of qualification loss was defined as the event date for all-cause resignation.
- Death (Component of Secondary Outcome and Censoring Event): If the reason for qualification loss was documented as "death" (死亡), this was classified as all-cause death. The date of qualification loss was:
  Treated as a censoring event for the primary outcome (resignation).
  Defined as an event date for the secondary composite outcome (resignation or death).
- Other Reasons for Loss of Qualification: If qualification was lost for reasons other than resignation or death (e.g., transition to a dependent status, unpaid premiums), individuals were censored at the date of such qualification loss.

It is important to note that the JHIA database does not allow for tracking individuals after they lose qualification from an employer at the index date, even if the resignation was for the purpose of changing jobs. If an individual subsequently gains employment at another JHIA-covered company, they would re-enroll in JHIA. However, this would be registered as a new qualification, and it is not possible to link this new enrollment to their previous JHIA coverage history. Therefore, "resignation" in this study is operationally defined as the cessation of employment (for any reason) from the company at which the individual was employed at the index date.

© 2025 Iwakura M et al. *JAMA Network Open*.

eAppendix 3. Details of the Covariate Definitions and Measurements

Demographics

Regarding the participants' demographics, we collected their age, residency region, classification of the affiliated entity, industrial classifications, monthly income, and years of service at the current workplace. First, the residency region was classified into seven regions: Hokkaido, Tohoku, Kanto, Chubu, Kinki, Chugoku/Shikoku, and Kyushu/Okinawa. Second, the classification of the affiliated entity was divided into two groups: corporate and individual. Third, the business type was divided into 18 categories based on the Health Insurance Act of Japan.[1] It included: Agriculture, forestry, and fisheries; Mining and quarrying of stone and gravel; Construction; Manufacturing; Electricity, gas, heat supply, and water; Information and communications; Transport and postal services; Wholesale and retail trade; Finance and insurance; Real estate and goods rental and leasing; Scientific research and professional and technical services; Accommodations and eating and drinking services; Living-related and personal services and amusement services; Education and learning support; Medical, healthcare, and welfare; Compound services; Services; and Government, except where classified. We combined Agriculture, forestry, and fisheries; Mining and quarrying of stone and gravel; Electricity, gas, heat supply and water; and Finance and insurance and classified them as "Others," due to the small number of women working in these respective business categories. Finally, monthly income data was obtained from the insurance premium profile, which recorded standardized monthly income data with 50 levels, ranging from level 1 (58,000 JPY) to level 50 (1,390,000 JPY).[2] We also dichotomized age, years of service, and monthly income by the median for analyses.

Results of the Health Check-up

The participants' BMI, alcohol consumption frequency, smoking status, and physical activity levels were obtained from their annual health check-up[3] results within the two-year lookback period. If an individual received multiple health checkups during the 2-year lookback period, then we selected the latest result.

First, BMI was categorized into three levels: less than 18.5 kg/m^2, 18.5 kg/m^2 to less than 25.0 kg/m^2, and 25.0 kg/m^2 or more. Second, alcohol drinking frequency included three categories: daily, sometimes, and hardly or never. Third, the smoking status was classified into two groups: current smokers and noncurrent smokers. Current smokers were defined as individuals who met the following criteria: 1) smoked at least 100 cigarettes in total or had a smoking habit longer than six months, and 2) smoked last month. Finally, physical activity levels were evaluated from two perspectives (i.e., engagement in light physical activity [LPA] and moderate to vigorous physical activity [MVPA]). Individuals were defined as engaged in LPA if they performed a walk or similar physical activity for one hour or more every day. Individuals were classified as engaged in MVPA if they met the following criteria: 1) exercised with moderate intensity two times a week or more; 2) exercised for 30 minutes or more in each session; and 3) continued their exercise habit for more than a year.

Past/Current Medical Histories

We collected data on depression (*ICD-10* codes, F32, F330–333, F338, F339, F341, and F412; positive predictive value [PPV] = 91%),[4] alcohol or drug abuse (*ICD-10* codes, E52, F10, G621, I426, K292, K700, K703, K709, T51, Z502, Z714, and Z721; two-year lookback, one hospitalization or two claims within the two-year lookback

© 2025 Iwakura M et al. *JAMA Network Open*.

period; PPV = 84%),[5] dementia (*ICD-10* codes, F00−F03, F051, G30, and G311; two-year lookback; one hospitalization or two claims within the two-year lookback period; PPV = 93%),[5, 6] and other cancers (C00–C26, C30–C34, C37–C41, C43–C49, C51, C52, C57, C58, C60–C86, C88, and C90–C97)[5, 6] as past/current medical histories. To ensure anonymization, we presented the data as <10 when the frequency of each cell was below this threshold.

Supplemental References

1. Ministry of Health, Labour and Welfare. Regarding the revision of the standard classification of business types for employees' health insurance and employees' pension insurance [Health Insurance Act]. https://www.mhlw.go.jp/web/t_doc?dataId=00tc6845&dataType=1&pageNo=1. Accessed September 12, 2024.
2. Japan Health Insurance Association. The amount of premium list every metropolis and districts. https://www.kyoukaikenpo.or.jp.e.ame.hp.transer.com/g3/cat330/sb3150/. Accessed September 13, 2024.
3. Japan Health Insurance Association. Medical examination, health instruction: What kind of inspection is there? https://www.kyoukaikenpo.or.jp.e.ame.hp.transer.com/g4/cat410/sb4020/. Accessed September 12, 2024.
4. Fiest KM, Jette N, Quan H, et al. Systematic review and assessment of validated case definitions for depression in administrative data. *BMC Psychiatry*. 2014;14:289.
5. Quan H, Sundararajan V, Halfon P, et al. Coding algorithms for defining comorbidities in ICD-9-CM and ICD-10 administrative data. *Medical Care*. 2005;43(11):1130.
6. Quan H, Li B, Duncan Saunders L, et al. Assessing validity of ICD-9-CM and ICD-10 administrative data in recording clinical conditions in a unique dually coded database. *Health Services Research*. 2008;43(4):1424-1441

© 2025 Iwakura M et al. *JAMA Network Open*.

eAppendix 4. Details of Missing Data Handling

Of the 13 covariates, 7 had missing values, with a missing proportion ranging from 0.01% to 54.6%. The missing values for monthly income and years of service at the workplace were minimal. The reason for these missing values was considered to be recording errors, and thus the mechanism for missing values was assumed to be missing completely at random (MCAR). In contrast, the proportion of missing values for BMI and other variables obtained from annual health checkup results was substantially higher. There are several reasons for the missing data in annual health checkup results. First, some of the missing values can be attributed to recording errors, which can also be considered MCAR. Additionally, the proportion of missing values varies by age, industry classification, and other covariates; these measured covariates may explain some of the missing data. Therefore, in this study, we assumed that the missing data mechanism was missing at random (MAR). We used multiple imputations to generate 300 imputed datasets with the "mice" package. Continuous variables were imputed using predictive mean matching, and categorical variables were imputed using logistic regression or multinomial logit models. The estimates from the imputed datasets were combined according to Rubin's rules.[1] For sensitivity analyses, unadjusted analyses were also performed.

Supplemental References

1. Toutenburg H. Rubin, DB.: Multiple imputation for nonresponse in surveys. *Stat Pap*. 1990;31(1):180.

© 2025 Iwakura M et al. *JAMA Network Open*.

eFigure 2. Cumulative Incidence Curves of Composite Outcome (Resignation or Death) After the Initial Cancer Diagnosis

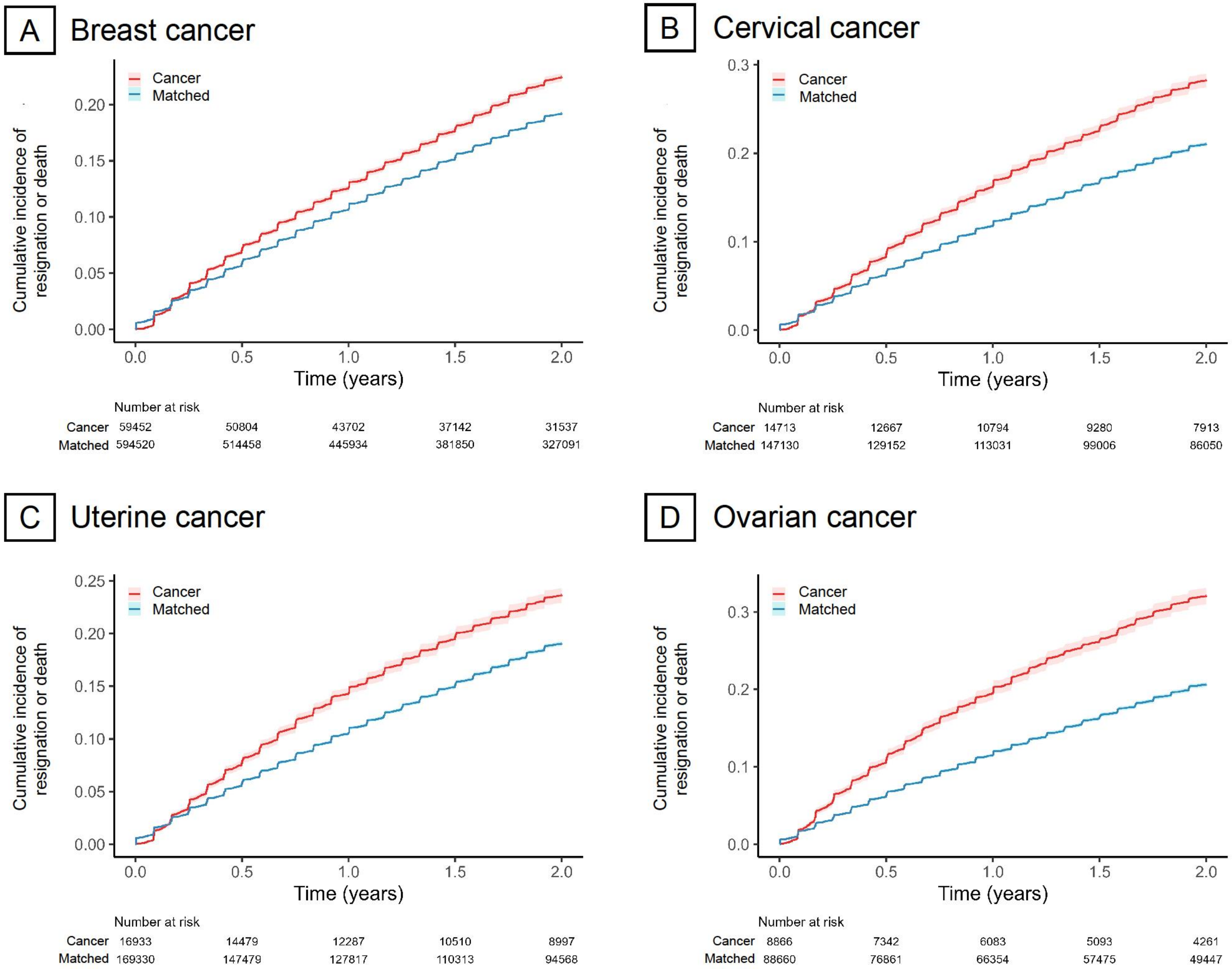

© 2025 Iwakura M et al. *JAMA Network Open*.

Each panel shows the cumulative incidence curve of the composite outcome (resignation or death) for women with breast cancer and matched individuals (A), with cervical cancer and matched individuals (B), with uterine cancer and matched individuals (C), and with ovarian cancer and matched individuals (D). The X-axis is the time (years) from the index date (women with cancer, the month of the initial cancer diagnosis; matched individuals, the index date of the corresponding patients). The Y-axis is the cumulative probability of resignation or death. The red line represents women with cancer, and the blue line represents matched individuals. The shaded area represents the 95% confidence intervals. The number of women with cancer and matched individuals followed up for each time interval is presented underneath each X-axis.

© 2025 Iwakura M et al. *JAMA Network Open*.

eFigure 3. Scaled Schoenfeld Residual Plots for Testing the Proportional-Hazards Assumption of the Primary Outcome

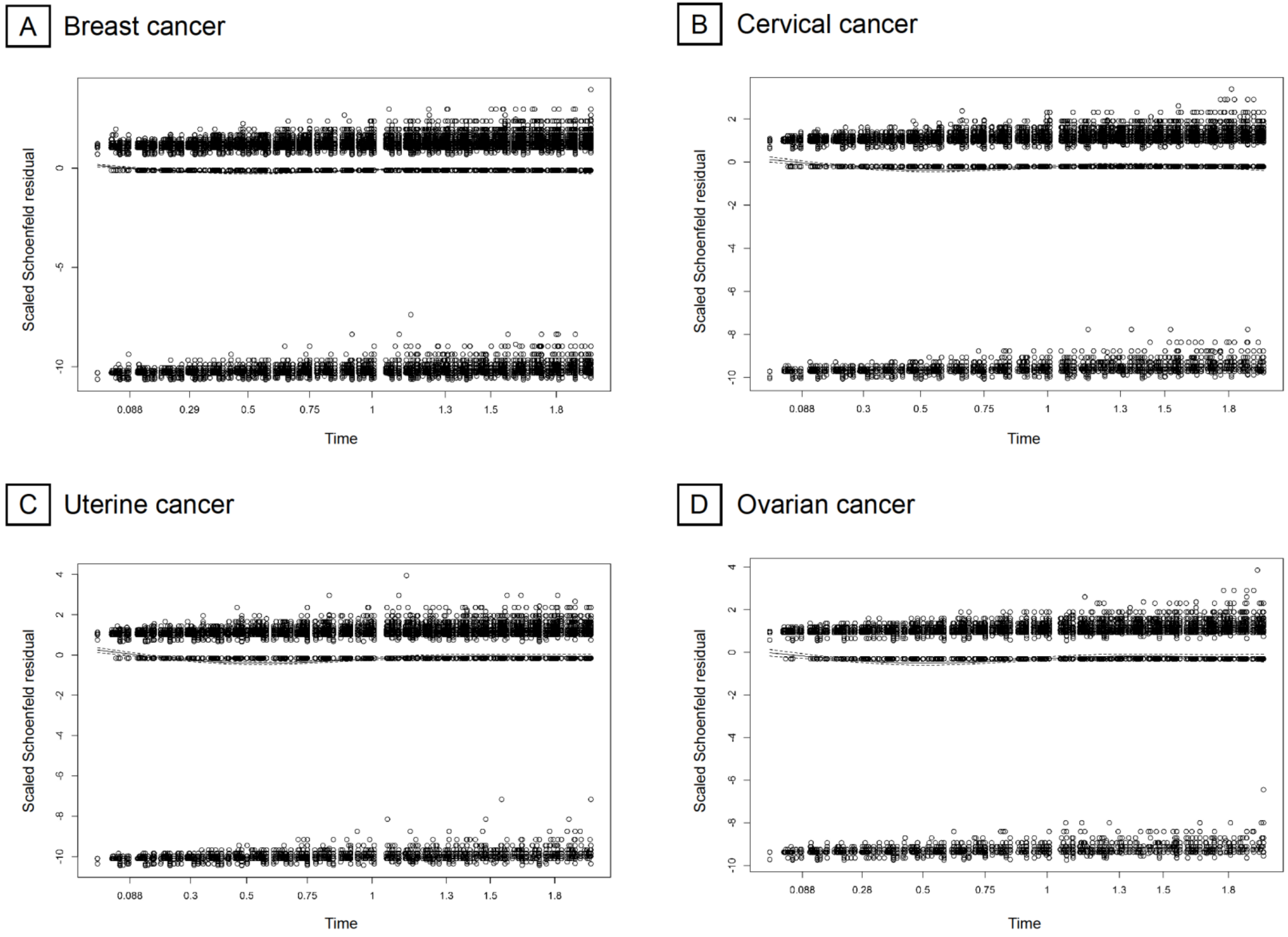

© 2025 Iwakura M et al. *JAMA Network Open*.

Each panel shows the scaled Schoenfeld residual plots of the primary outcome for women with breast cancer and matched individuals (A), with cervical cancer and matched individuals (B), with uterine cancer and matched individuals (C), and with ovarian cancer and matched individuals (D). The X-axis is the time (years) from the index date (women with cancer, the month of the initial cancer diagnosis; matched individuals, the index date of the corresponding woman with cancer). The Y-axis is the scaled Schoenfeld residual. Each dot represents the residual at an event time. The solid lines are smoothed loess curves fitted to the residuals. The dashed lines represent the 95% confidence intervals. A flat loess curve suggests that the proportional hazard assumption is satisfied for each exposure. The Grambsch and Therneau's score test also suggests that there is no evidence of the serious violation of the assumption of proportional hazards for all cohorts (P = 0.600 for breast cancer, P = 0.570 for cervical cancer, P = 0.20 for uterine body cancer, and P = 0.130 for ovarian cancer).

© 2025 Iwakura M et al. *JAMA Network Open*.

eFigure 4. Scaled Schoenfeld Residual Plots for Testing the Proportional-Hazards Assumption of the Secondary Outcome

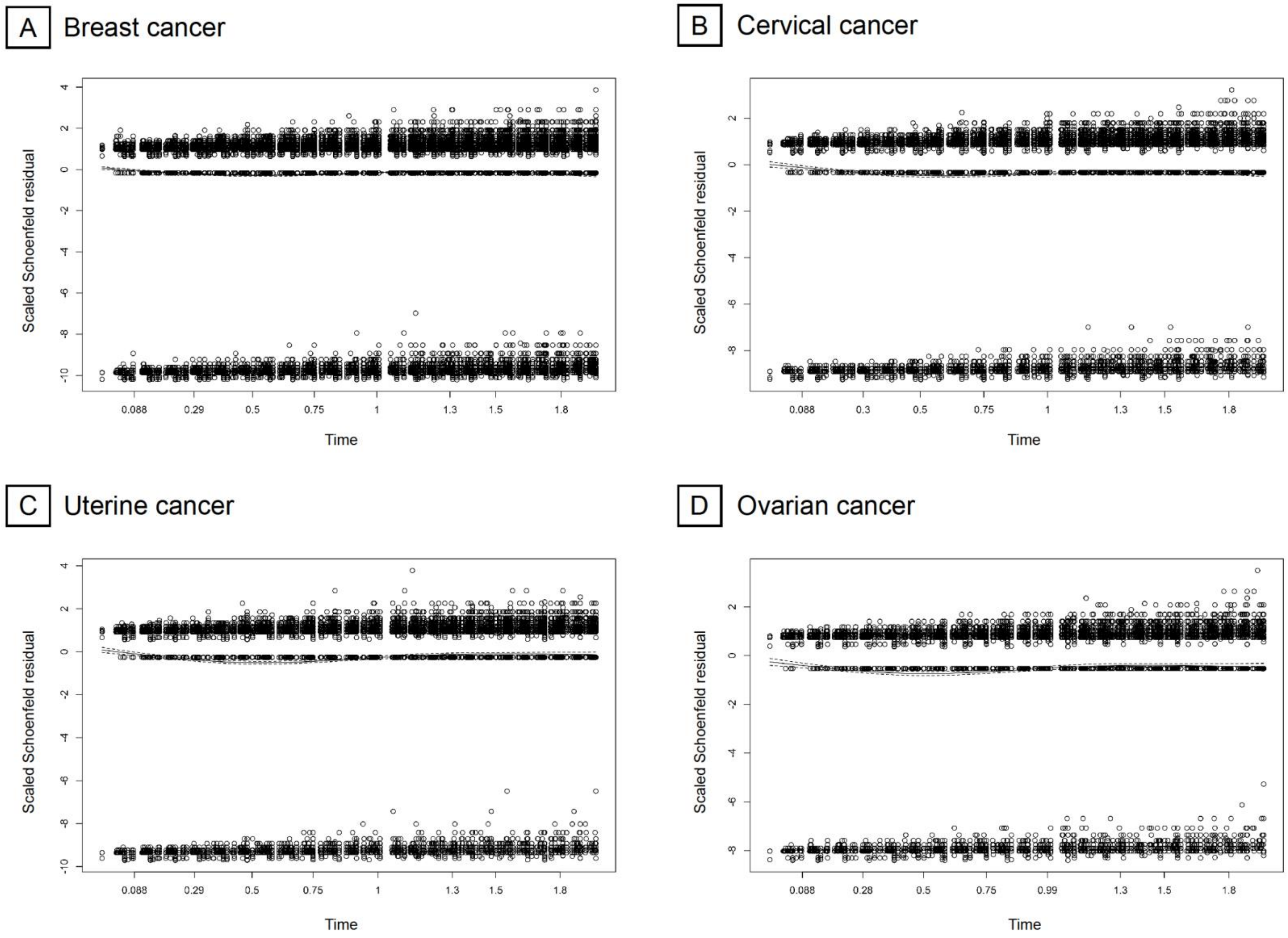

© 2025 Iwakura M et al. *JAMA Network Open*.

Each panel shows the scaled Schoenfeld residual plots of the secondary outcome (resignation or death) for women with breast cancer and matched individuals (A), with cervical cancer and matched individuals (B), with uterine cancer and matched individuals (C), and with ovarian cancer and matched individuals (D). The X-axis is the time (years) from the index date (women with cancer, the month of the initial cancer diagnosis; matched individuals, the index date of the corresponding woman with cancer). The Y-axis is the scaled Schoenfeld residual. Each dot represents the residual at an event time. The solid lines are smoothed loess curves fitted to the residuals. The dashed lines represent the 95% confidence intervals. A flat loess curve suggests that the proportional hazard assumption is satisfied for each exposure. The Grambsch and Therneau's score test also suggests that there is no evidence of a serious violation of the assumption of proportional hazards for the three cohorts (P = 0.150 for breast cancer, P = 0.060 for cervical cancer, and P = 0.110 for ovarian cancer); while not for uterine body cancer (P = 0.006).

© 2025 Iwakura M et al. *JAMA Network Open*.

eFigure 5. Subgroup analyses for the Associations Between Initial Diagnosis of Breast Cancer and Resignation in 13 Prespecified Covariates

Variable	Level	Matched N (Event)	Cancer N (Event)	HR (95% CI)	P for interaction
Age	< median	297110 (52268)	29691 (5548)	1.11 (1.08, 1.14)	<.001
	>= median	297410 (45624)	29761 (5272)	1.22 (1.19, 1.26)	
Residency region	Hokkaido	25255 (4377)	2760 (567)	1.27 (1.16, 1.39)	.33
	Tohoku	50743 (7205)	4740 (763)	1.16 (1.08, 1.26)	
	Kanto	157960 (28839)	16566 (3199)	1.14 (1.10, 1.18)	
	Chubu	115834 (17746)	11096 (1873)	1.17 (1.12, 1.23)	
	Kinki	96468 (16038)	10163 (1872)	1.14 (1.09, 1.20)	
	Chugoku/Shikoku	66448 (10021)	6040 (1025)	1.17 (1.10, 1.25)	
	Kyushu/Okinawa	81812 (13666)	8087 (1521)	1.19 (1.13, 1.25)	
Monthly income	< median	251027 (48194)	22806 (5175)	1.26 (1.22, 1.30)	<.001
	>= median	343264 (49607)	36646 (5645)	1.08 (1.05, 1.11)	
Years of service	< median	297437 (65840)	29434 (7055)	1.13 (1.10, 1.15)	.78
	>= median	296854 (31961)	30018 (3765)	1.20 (1.16, 1.24)	
Affiliated entity classification: corporate	No	15076 (2542)	1567 (318)	1.26 (1.12, 1.42)	.23
	Yes	579444 (95350)	57885 (10502)	1.16 (1.14, 1.18)	
Industrial classification	Construction	26772 (2892)	2960 (330)	1.05 (0.94, 1.18)	<.001
	Manifacturing	81791 (10684)	7798 (1154)	1.18 (1.11, 1.25)	
	Inofrmation	7610 (1197)	985 (144)	0.95 (0.79, 1.13)	
	Transportation	17271 (2615)	1688 (281)	1.12 (0.99, 1.27)	
	Wholesale	81434 (12315)	8524 (1487)	1.19 (1.13, 1.25)	
	Real estate	13182 (1938)	1581 (200)	0.90 (0.78, 1.04)	
	Scientific research	24094 (3703)	2925 (469)	1.07 (0.97, 1.18)	
	Accomodations	17170 (3685)	1498 (348)	1.14 (1.02, 1.28)	
	Living related service	17776 (3354)	1681 (281)	0.91 (0.81, 1.03)	
	Education	11869 (2658)	1276 (320)	1.17 (1.04, 1.32)	
	Medical/Welfare	194909 (31965)	18754 (3556)	1.20 (1.16, 1.24)	
	Compound services	8215 (1045)	816 (119)	1.25 (1.03, 1.51)	
	Services	56701 (12600)	5490 (1264)	1.19 (1.12, 1.26)	
	Goverment	25233 (5855)	2383 (715)	1.40 (1.30, 1.52)	
	Others	10493 (1386)	1093 (152)	1.10 (0.93, 1.30)	
Health checkup history	No	223067 (42069)	19285 (4018)	1.21 (1.17, 1.25)	.02
	Yes	371453 (55823)	40167 (6802)	1.15 (1.12, 1.18)	
BMI	< 18.5	46332 (7465)	4921 (897)	1.13 (1.06, 1.21)	.05
	< 25	243741 (36132)	26924 (4528)	1.16 (1.13, 1.20)	
	>= 25	81380 (12226)	8322 (1377)	1.13 (1.07, 1.19)	
Alcohol drinking frequency	Hardly/Never	169279 (24861)	17710 (2950)	1.17 (1.12, 1.21)	.66
	Sometimes	97666 (14854)	10802 (1831)	1.13 (1.08, 1.19)	
	Everyday	54228 (8052)	6435 (1066)	1.14 (1.07, 1.22)	
Smoking status	No	304900 (44432)	33348 (5492)	1.15 (1.12, 1.19)	.59
	Yes	62277 (10385)	6314 (1196)	1.17 (1.10, 1.24)	
Engagement in MV PA	No	272764 (40289)	29768 (4944)	1.15 (1.12, 1.18)	.92
	Yes	45141 (6977)	4881 (840)	1.15 (1.07, 1.24)	
Engagement in L PA	No	208362 (29624)	23031 (3640)	1.13 (1.09, 1.17)	.56
	Yes	109699 (17678)	11635 (2153)	1.18 (1.13, 1.24)	
History of depression	No	590826 (97469)	56768 (10222)	1.14 (1.12, 1.17)	<.001
	Yes	3694 (423)	2684 (598)	2.16 (1.91, 2.45)	

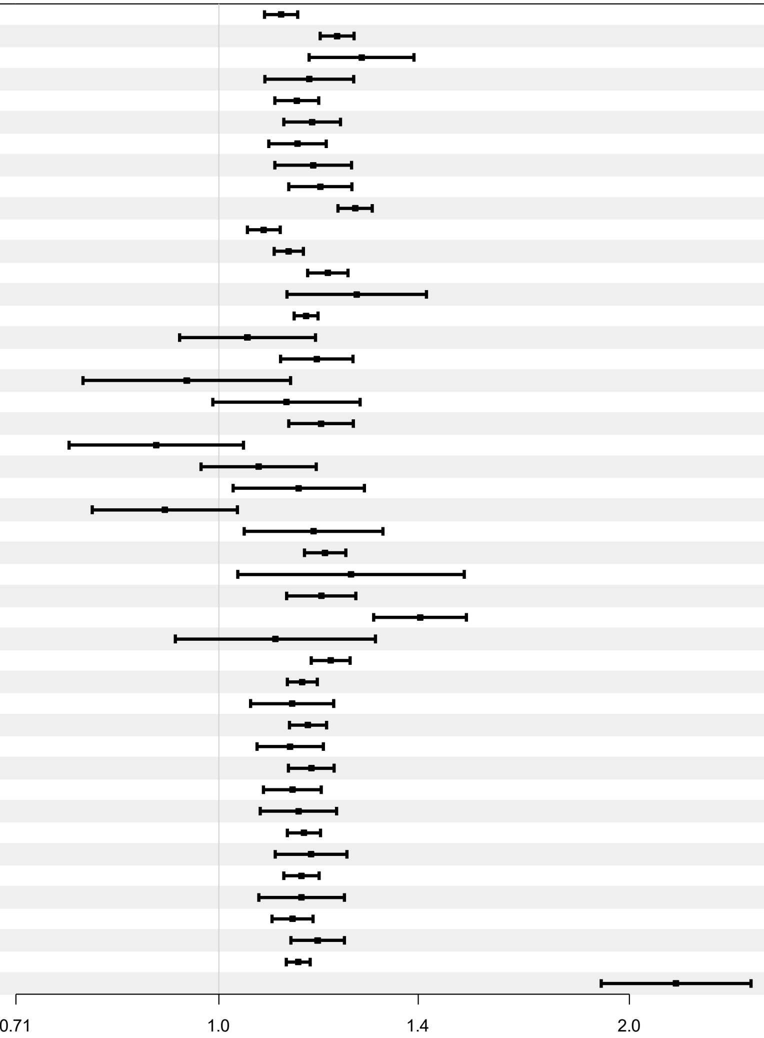

© 2025 Iwakura M et al. *JAMA Network Open*.

This figure represents the results of prespecified subgroup analyses on the association between the initial diagnosis of breast cancer and resignation using Cox proportional-hazards models, including the interaction term of one subgroup and the incidence of each cancer. All subgroup analyses were performed with imputed datasets and adjustments on the same covariates (except for each subgroup). Covariates used for adjustments in the analysis were the following: age, residency region, classification of the affiliated entity, industrial classification, monthly income, year of service, health checkup history, BMI, alcohol drinking frequency, smoking status, physical activity (engagement in MVPA and engagement in LPA), and history of depression. Industrial classification comprised 15 categories: Construction; Manufacturing; Information and communications; Transport and postal services; Wholesale and retail trade; Real estate and goods rental and leasing; Scientific research, professional and technical services; accommodation, eating and drinking services; Living-related and personal services and amusement services; Education, learning support; Medical, health care and welfare; Compound services; Services; Government, except elsewhere classified; Others, Combination of Agriculture, forestry, and fisheries, Mining and quarrying of stone and gravel, Electricity, gas, heat supply and water, and Finance and insurance. The dots represent point estimates, and the error bars imply 95% CIs.

Abbreviations: BMI, body mass index; CI, confidence interval; HR, hazard ratio; LPA, light intensity physical activity; MVPA, moderate to vigorous physical activity.

© 2025 Iwakura M et al. *JAMA Network Open*.

eFigure 6. Subgroup analyses for the Associations Between Initial Diagnosis of Cervical Cancer and Resignation in 13 Prespecified Covariates

Variable	Level	Matched N (Event)	Cancer N (Event)	HR (95% CI)	P for interaction
Age	< median	73444 (15845)	7333 (1794)	1.20 (1.14, 1.26)	<.001
	>= median	73686 (11631)	7380 (1502)	1.39 (1.31, 1.46)	
Residency region	Hokkaido	6209 (1188)	605 (151)	1.47 (1.24, 1.75)	.04
	Tohoku	12620 (2075)	1210 (244)	1.25 (1.09, 1.43)	
	Kanto	39805 (8263)	3665 (886)	1.27 (1.18, 1.36)	
	Chubu	28127 (4940)	2217 (483)	1.36 (1.24, 1.50)	
	Kinki	24078 (4536)	2258 (488)	1.23 (1.12, 1.36)	
	Chugoku/Shi koku	16046 (2664)	1525 (335)	1.41 (1.25, 1.58)	
	Kyushu/Okin awa	20245 (3810)	3233 (709)	1.15 (1.06, 1.25)	
Monthly income	< median	64322 (13832)	6187 (1682)	1.35 (1.28, 1.42)	.03
	>= median	82763 (13625)	8526 (1614)	1.19 (1.12, 1.25)	
Years of se rvice	< median	73569 (18733)	7322 (2072)	1.15 (1.10, 1.20)	<.001
	>= median	73516 (8724)	7391 (1224)	1.46 (1.38, 1.55)	
Affiliated entity classification: co rporate	No	3813 (708)	389 (91)	1.27 (1.00, 1.60)	.76
	Yes	143317 (26768)	14324 (3205)	1.27 (1.22, 1.32)	
Industrial classification	Construction	6246 (733)	723 (91)	1.13 (0.90, 1.41)	.16
	Manifacturing	19710 (3070)	1884 (357)	1.27 (1.14, 1.42)	
	Inofrmation	2098 (389)	192 (36)	1.04 (0.73, 1.49)	
	Transportation	4077 (647)	448 (105)	1.49 (1.20, 1.85)	
	Wholesale	20257 (3525)	2092 (446)	1.26 (1.14, 1.40)	
	Real estate	3328 (573)	374 (70)	1.06 (0.82, 1.38)	
	Scientific research	6240 (1158)	552 (116)	1.24 (1.02, 1.51)	
	Accomodations	4281 (1026)	481 (122)	1.19 (0.98, 1.45)	
	Living related se rvice	4979 (1165)	620 (148)	1.05 (0.88, 1.25)	
	Education	3204 (849)	296 (85)	1.14 (0.91, 1.43)	
	Medical/Welfare	48419 (8756)	4978 (1148)	1.33 (1.24, 1.41)	
	Compound services	1855 (261)	206 (35)	1.37 (0.95, 1.97)	
	Services	13841 (3393)	1225 (346)	1.29 (1.15, 1.44)	
	Goverment	6050 (1534)	407 (147)	1.49 (1.25, 1.77)	
	Others	2545 (397)	235 (44)	1.28 (0.93, 1.77)	
Health checkup histo ry	No	66530 (14602)	6472 (1602)	1.26 (1.19, 1.33)	.13
	Yes	80600 (12874)	8241 (1694)	1.31 (1.25, 1.38)	
BMI	< 18.5	12517 (2239)	1370 (294)	1.22 (1.08, 1.38)	<.001
	< 25	51463 (8012)	5322 (1089)	1.34 (1.25, 1.43)	
	>= 25	16620 (2623)	1549 (311)	1.32 (1.17, 1.49)	
Alcohol d rinking frequency	Hardly/Never	35447 (5550)	3333 (709)	1.41 (1.30, 1.52)	.46
	Sometimes	21006 (3305)	2189 (430)	1.24 (1.12, 1.37)	
	Everyday	11047 (1775)	1357 (262)	1.24 (1.09, 1.41)	
Smoking status	No	63971 (9689)	5977 (1171)	1.34 (1.26, 1.43)	.83
	Yes	13432 (2432)	1853 (421)	1.31 (1.18, 1.45)	
Engagement in MV PA	No	57425 (9019)	5875 (1168)	1.29 (1.21, 1.37)	.15
	Yes	9345 (1507)	934 (222)	1.53 (1.33, 1.77)	
Engagement in L PA	No	43606 (6564)	4418 (868)	1.34 (1.25, 1.44)	.76
	Yes	23177 (3961)	2401 (523)	1.28 (1.17, 1.41)	
History of depression	No	146327 (27372)	13980 (3093)	1.25 (1.20, 1.30)	<.001
	Yes	803 (104)	733 (203)	2.48 (1.93, 3.18)	

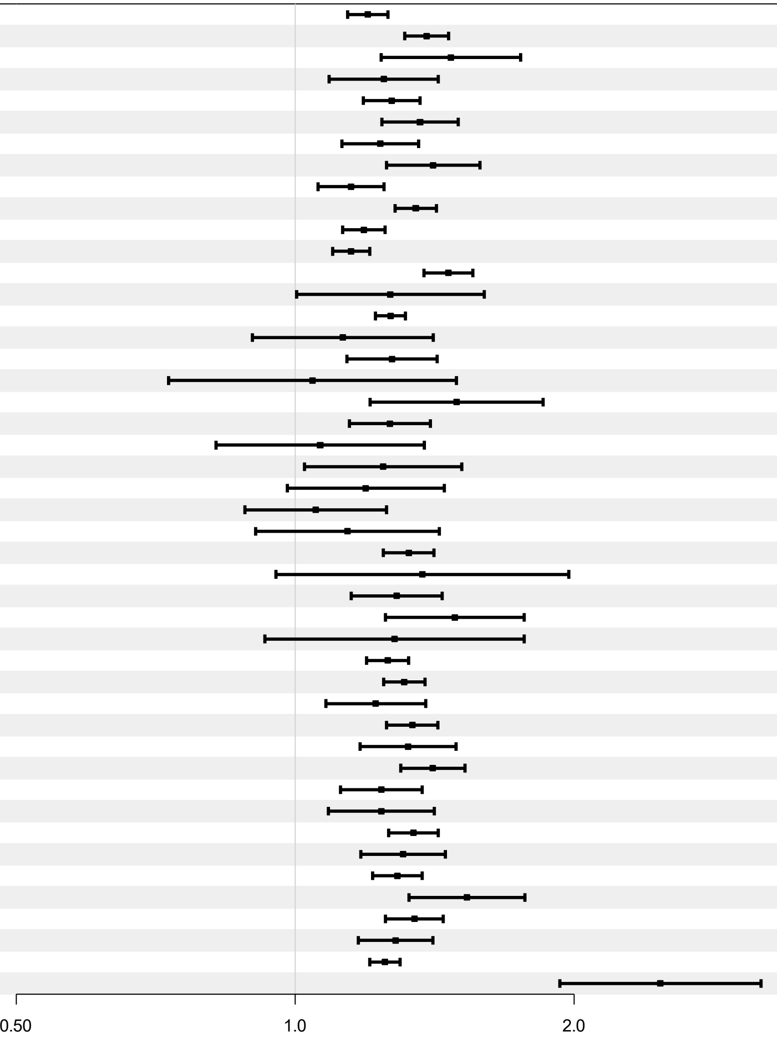

© 2025 Iwakura M et al. *JAMA Network Open*.

This figure represents the results of prespecified subgroup analyses on the association between the initial diagnosis of cervical cancer and resignation using Cox proportional-hazards models, including the interaction term of one subgroup and the incidence of each cancer. All subgroup analyses were performed with imputed datasets and adjustments on the same covariates (except for each subgroup). Covariates used for adjustments in the analysis were the following: age, residency region, classification of the affiliated entity, industrial classification, monthly income, year of service, health checkup history, BMI, alcohol drinking frequency, smoking status, physical activity (engagement in MVPA and engagement in LPA), and history of depression. Industrial classification comprised 15 categories: Construction; Manufacturing; Information and communications; Transport and postal services; Wholesale and retail trade; Real estate and goods rental and leasing; Scientific research, professional and technical services; accommodation, eating and drinking services; Living-related and personal services and amusement services; Education, learning support; Medical, health care and welfare; Compound services; Services; Government, except elsewhere classified; Others, Combination of Agriculture, forestry, and fisheries, Mining and quarrying of stone and gravel, Electricity, gas, heat supply and water, and Finance and insurance. The dots represent point estimates, and the error bars imply 95% CIs.

Abbreviations: BMI, body mass index; CI, confidence interval; HR, hazard ratio; LPA, light intensity physical activity; MVPA, moderate to vigorous physical activity.

© 2025 Iwakura M et al. *JAMA Network Open*.

eFigure 7. Subgroup analyses for the Associations between the Initial Diagnosis of Uterine Cancer and Resignation in 13 Prespecified Covariates

Variable	Level	Matched N (Event)	Cancer N (Event)	HR (95% CI)	P for interaction
Age	< median	84112 (15095)	8314 (1616)	1.16 (1.10, 1.23)	.002
	>= median	85218 (12691)	8619 (1545)	1.28 (1.21, 1.35)	
Residency region	Hokkaido	7224 (1265)	746 (144)	1.19 (1.00, 1.42)	.58
	Tohoku	14485 (2083)	1272 (210)	1.22 (1.05, 1.41)	
	Kanto	44525 (8164)	4885 (990)	1.21 (1.13, 1.30)	
	Chubu	33006 (4966)	2761 (473)	1.25 (1.14, 1.38)	
	Kinki	27556 (4578)	2499 (453)	1.18 (1.07, 1.30)	
	Chugoku/Shikoku	18836 (2857)	1925 (360)	1.34 (1.19, 1.50)	
	Kyushu/Okinawa	23698 (3873)	2845 (531)	1.16 (1.06, 1.27)	
Monthly income	< median	71633 (13620)	6985 (1562)	1.26 (1.20, 1.33)	.01
	>= median	97645 (14148)	9948 (1599)	1.17 (1.11, 1.23)	
Years of service	< median	84681 (18936)	8407 (2010)	1.12 (1.07, 1.17)	<.001
	>= median	84597 (8832)	8526 (1151)	1.37 (1.28, 1.46)	
Affiliated entity classification: corporate	No	4212 (725)	464 (92)	1.17 (0.94, 1.47)	.73
	Yes	165118 (27061)	16469 (3069)	1.22 (1.17, 1.26)	
Industrial classification	Construction	7705 (785)	773 (84)	1.04 (0.82, 1.30)	.35
	Manifacturing	23378 (2971)	2312 (341)	1.22 (1.09, 1.37)	
	Inofrmation	2218 (378)	240 (52)	1.65 (1.22, 2.24)	
	Transportation	4819 (696)	489 (75)	1.10 (0.86, 1.41)	
	Wholesale	23337 (3539)	2411 (453)	1.36 (1.23, 1.50)	
	Real estate	3638 (508)	372 (49)	1.04 (0.77, 1.42)	
	Scientific research	6781 (1050)	727 (114)	1.05 (0.86, 1.29)	
	Accomodations	4946 (1089)	406 (113)	1.42 (1.16, 1.73)	
	Living related service	5090 (991)	457 (97)	1.09 (0.88, 1.35)	
	Education	3338 (745)	324 (82)	1.24 (0.98, 1.57)	
	Medical/Welfare	55755 (9176)	5559 (1042)	1.18 (1.11, 1.26)	
	Compound services	2348 (274)	288 (35)	1.11 (0.77, 1.61)	
	Services	15824 (3473)	1526 (361)	1.20 (1.07, 1.34)	
	Goverment	7156 (1696)	717 (212)	1.34 (1.16, 1.56)	
	Others	2997 (415)	332 (51)	1.20 (0.89, 1.61)	
Health checkup history	No	65505 (12552)	6226 (1300)	1.17 (1.11, 1.24)	.36
	Yes	103825 (15234)	10707 (1861)	1.23 (1.17, 1.29)	
BMI	< 18.5	13617 (2121)	1062 (184)	1.11 (0.95, 1.29)	.14
	< 25	67546 (9861)	6135 (1062)	1.21 (1.14, 1.29)	
	>= 25	22662 (3252)	3510 (615)	1.29 (1.18, 1.40)	
Alcohol drinking frequency	Hardly/Never	46766 (6706)	5031 (847)	1.22 (1.14, 1.31)	.78
	Sometimes	27129 (3984)	2816 (485)	1.21 (1.10, 1.33)	
	Everyday	15270 (2259)	1358 (235)	1.17 (1.02, 1.34)	
Smoking status	No	84895 (11986)	9025 (1545)	1.24 (1.18, 1.31)	.23
	Yes	17143 (2863)	1518 (276)	1.12 (0.99, 1.27)	
Engagement in MV PA	No	75593 (10984)	7803 (1308)	1.19 (1.12, 1.26)	.19
	Yes	12577 (1811)	1314 (247)	1.38 (1.20, 1.58)	
Engagement in L PA	No	57775 (8004)	6030 (996)	1.22 (1.14, 1.30)	.86
	Yes	30407 (4799)	3076 (555)	1.21 (1.11, 1.32)	
History of depression	No	168266 (27670)	16059 (2959)	1.19 (1.15, 1.24)	<.001
	Yes	1064 (116)	874 (202)	2.30 (1.81, 2.92)	

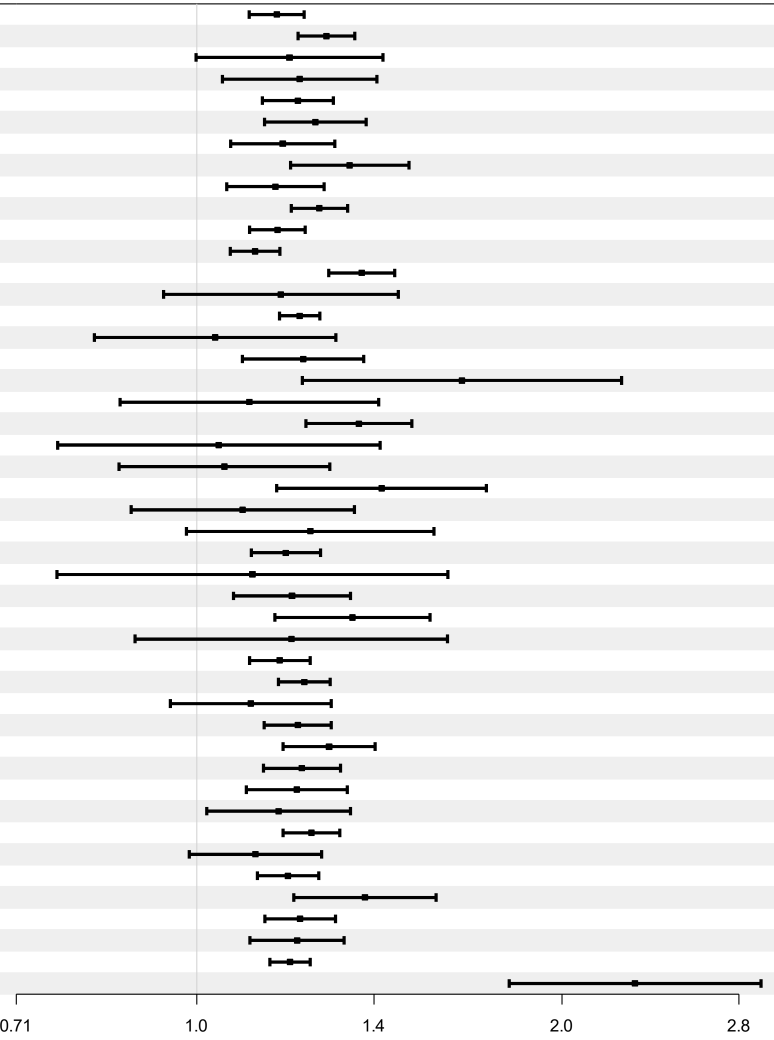

© 2025 Iwakura M et al. *JAMA Network Open*.

This figure represents the results of prespecified subgroup analyses on the association between the initial diagnosis of uterine cancer and resignation using Cox proportional-hazards models, including the interaction term of one subgroup and the incidence of each cancer. All subgroup analyses were performed with imputed datasets and adjustments on the same covariates (except for each subgroup). Covariates used for adjustments in the analysis were the following: age, residency region, classification of the affiliated entity, industrial classification, monthly income, year of service, health checkup history, BMI, alcohol drinking frequency, smoking status, physical activity (engagement in MVPA and engagement in LPA), and history of depression. Industrial classification comprised 15 categories: Construction; Manufacturing; Information and communications; Transport and postal services; Wholesale and retail trade; Real estate and goods rental and leasing; Scientific research, professional and technical services; accommodation, eating and drinking services; Living-related and personal services and amusement services; Education, learning support; Medical, health care and welfare; Compound services; Services; Government, except elsewhere classified; Others, Combination of Agriculture, forestry, and fisheries, Mining and quarrying of stone and gravel, Electricity, gas, heat supply and water, and Finance and insurance. The dots represent point estimates, and the error bars imply 95% CIs.

Abbreviations: BMI, body mass index; CI, confidence interval; HR, hazard ratio; LPA, light intensity physical activity; MVPA, moderate to vigorous physical activity.

© 2025 Iwakura M et al. *JAMA Network Open*.

eFigure 8. Subgroup analyses for the Associations Between Initial Diagnosis of Ovarian Cancer and Resignation in 13 Prespecified Covariates

Variable	Level	Matched N (Event)	Cancer N (Event)	HR (95% CI)	P for interaction
Age	< median	44322 (9093)	4428 (1035)	1.28 (1.20, 1.37)	<.001
	>= median	44338 (6754)	4438 (969)	1.67 (1.55, 1.79)	
Residency region	Hokkaido	3734 (718)	274 (69)	1.03 (0.49, 2.17)	.55
	Tohoku	7423 (1164)	712 (143)	1.08 (0.76, 1.53)	
	Kanto	23530 (4633)	2538 (603)	1.41 (1.27, 1.57)	
	Chubu	17330 (2853)	1582 (345)	1.61 (1.37, 1.89)	
	Kinki	14513 (2605)	1494 (318)	1.30 (1.09, 1.54)	
	Chugoku/Shikoku	9786 (1620)	1052 (246)	1.29 (1.01, 1.64)	
	Kyushu/Okinawa	12344 (2254)	1214 (280)	1.48 (1.22, 1.80)	
Monthly income	< median	37547 (7750)	3550 (986)	1.66 (1.53, 1.79)	<.001
	>= median	51088 (8084)	5316 (1018)	1.31 (1.22, 1.41)	
Years of service	< median	44341 (10807)	4406 (1270)	1.28 (1.20, 1.36)	.01
	>= median	44294 (5027)	4460 (734)	1.56 (1.44, 1.70)	
Affiliated entity classification: corporate	No	2270 (441)	232 (61)	1.47 (0.54, 4.00)	.90
	Yes	86390 (15406)	8634 (1943)	1.44 (1.37, 1.51)	
Industrial classification	Construction	3823 (444)	364 (49)	1.64 (0.75, 3.59)	.60
	Manifacturing	12062 (1714)	1214 (225)	1.67 (1.34, 2.08)	
	Inofrmation	1195 (247)	155 (27)	1.29 (0.08, 20.09)	
	Transportation	2534 (368)	278 (58)	2.02 (0.82, 5.02)	
	Wholesale	12260 (2073)	1320 (296)	1.51 (1.24, 1.84)	
	Real estate	1993 (277)	222 (35)	0.57 (0.09, 3.58)	
	Scientific research	3719 (648)	418 (91)	1.16 (0.67, 2.02)	
	Accomodations	2621 (618)	235 (61)	1.46 (0.66, 3.23)	
	Living related service	2863 (601)	311 (92)	1.19 (0.63, 2.27)	
	Education	1830 (406)	189 (57)	0.62 (0.17, 2.25)	
	Medical/Welfare	29195 (5227)	2832 (652)	1.42 (1.28, 1.56)	
	Compound services	1112 (138)	118 (22)	2.05 (1.28, 3.28)	
	Services	8337 (1936)	748 (193)	1.60 (1.24, 2.06)	
	Goverment	3514 (911)	323 (114)	2.13 (1.27, 3.58)	
	Others	1602 (239)	139 (32)	1.58 (1.07, 2.32)	
Health checkup history	No	38110 (8089)	3608 (899)	1.37 (1.27, 1.48)	.09
	Yes	50550 (7758)	5258 (1105)	1.47 (1.37, 1.57)	
BMI	< 18.5	7539 (1307)	764 (185)	1.71 (1.27, 2.31)	.06
	< 25	32320 (4861)	3225 (673)	1.47 (1.34, 1.61)	
	>= 25	10691 (1590)	1269 (247)	1.48 (1.20, 1.83)	
Alcohol drinking frequency	Hardly/Never	22626 (3354)	2448 (494)	1.44 (1.28, 1.62)	.94
	Sometimes	12954 (2004)	1307 (275)	1.34 (1.11, 1.61)	
	Everyday	7090 (1046)	659 (143)	1.59 (1.13, 2.26)	
Smoking status	No	40426 (5925)	4251 (881)	1.53 (1.41, 1.65)	.19
	Yes	8362 (1394)	791 (153)	1.21 (0.92, 1.59)	
Engagement in MV PA	No	36273 (5393)	3753 (755)	1.46 (1.34, 1.59)	.57
	Yes	5882 (933)	619 (147)	1.80 (1.24, 2.61)	
Engagement in L PA	No	27559 (4000)	2862 (570)	1.41 (1.27, 1.56)	.71
	Yes	14617 (2322)	1500 (329)	1.57 (1.32, 1.85)	
History of depression	No	88166 (15789)	8458 (1897)	1.43 (1.36, 1.50)	.03
	Yes	494 (58)	408 (107)	2.41 (1.70, 3.41)	

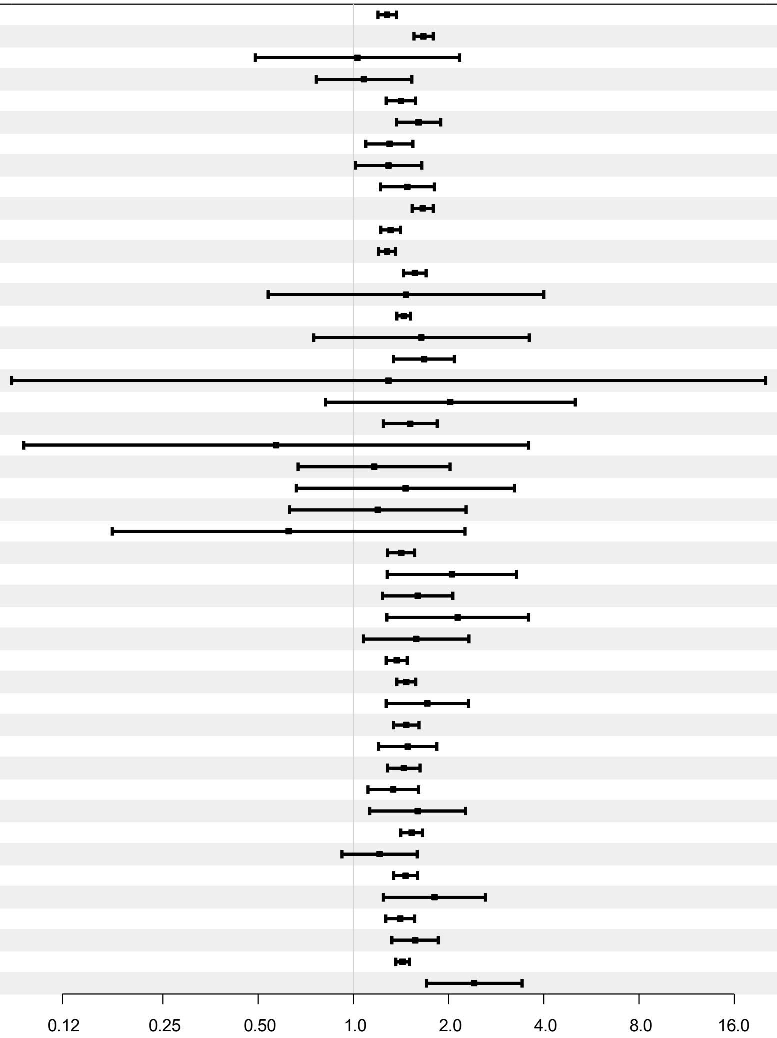

© 2025 Iwakura M et al. *JAMA Network Open*.

This figure represents the results of prespecified subgroup analyses on the association between the initial diagnosis of ovarian cancer and resignation using Cox proportional-hazards models, including the interaction term of one subgroup and the incidence of each cancer. All subgroup analyses were performed with imputed datasets and adjustments on the same covariates (except for each subgroup). Covariates used for adjustments in the analysis were the following: age, residency region, classification of the affiliated entity, industrial classification, monthly income, year of service, health checkup history, BMI, alcohol drinking frequency, smoking status, physical activity (engagement in MVPA and engagement in LPA), and history of depression. Industrial classification comprised 15 categories: Construction; Manufacturing; Information and communications; Transport and postal services; Wholesale and retail trade; Real estate and goods rental and leasing; Scientific research, professional and technical services; accommodation, eating and drinking services; Living-related and personal services and amusement services; Education, learning support; Medical, health care and welfare; Compound services; Services; Government, except elsewhere classified; Others, Combination of Agriculture, forestry, and fisheries, Mining and quarrying of stone and gravel, Electricity, gas, heat supply and water, and Finance and insurance. The dots represent point estimates, and the error bars imply 95% CIs.

Abbreviations: BMI, body mass index; CI, confidence interval; HR, hazard ratio; LPA, light intensity physical activity; MVPA, moderate to vigorous physical activity.

© 2025 Iwakura M et al. *JAMA Network Open*.

eTable 5. Results of an Additional Sensitivity Analysis Stratified by Fiscal Year of Index Date to Assess the Potential Impact of the COVID-19 Pandemic on the Association between Initial Diagnosis of Cancers and Resignation

Primary outcome:	Index date	Cohort 1: Breast cancer		Cohort 2: Cervical cancer		Cohort 3 Uterine cancer		Cohort 4: Ovarian cancer	
Resignation	(Fiscal year)	Cancer	Matched	Cancer	Matched	Cancer	Matched	Cancer	Matched
Crude resignation rate (%)	2016	19.6	17.8	24.2	19.9	20.7	17.5	23.7	18.9
	2017	19.0	17.8	23.0	19.6	19.2	17.3	25.2	18.6
	2018	19.8	18.2	23.9	19.9	20.0	18.5	25.4	19.0
	2019	19.5	17.5	22.0	19.5	19.4	17.2	24.9	19.1
	2020	18.9	16.5	22.5	18.4	18.7	16.7	21.6	18.1
	2021	20.3	18.0	25.8	20.6	20.5	17.6	23.7	19.8
Adjusted HR [95%CI]	2016	1.185 [1.114, 1.260]		1.350 [1.228, 1.484]		1.319 [1.180, 1.476]		1.461 [1.268, 1.682]	
	2017	1.155 [1.093, 1.221]		1.284 [1.173, 1.406]		1.229 [1.107, 1.364]		1.615 [1.426, 1.830]	
	2018	1.168 [1.106, 1.234]		1.308 [1.183, 1.445]		1.156 [1.044, 1.281]		1.561 [1.375, 1.771]	
	2019	1.198 [1.137, 1.263]		1.212 [1.090, 1.347]		1.226 [1.109, 1.356]		1.528 [1.344, 1.738]	
	2020	1.233 [1.168, 1.302]		1.362 [1.220, 1.519]		1.236 [1.109, 1.377]		1.353 [1.185, 1.544]	
	2021	1.201 [1.144, 1.261]		1.381 [1.255, 1.519]		1.263 [1.152, 1.386]		1.356 [1.200, 1.531]	

All adjusted models used matched individuals as references. The results presented are based on multiple imputation for missing covariates. “Adjusted” implies that the model was adjusted for age, residency region, classification of the affiliated entity, industrial classifications, monthly income, year of service, health checkup history, body mass index, alcohol drinking frequency, smoking status, physical activity (engagement in moderate to vigorous physical activity and engagement in light physical activity), and history of depression.

Abbreviations: CI, confidence interval; HR, hazard ratio.

© 2025 Iwakura M et al. *JAMA Network Open*.
