# Supplementary material for: Resignation in Working Women With Breast and Gynecologic Cancers
Source: JAMA Netw Open. 2025 Aug 25;8(8):e2528844. doi: 10.1001/jamanetworkopen.2025.28844 (PMC12379106; doi:10.1001/jamanetworkopen.2025.28844)
Supplement: Supplement 2. — Data Sharing Statement [file jamanetwopen-e2528844-s002.pdf]

# Data Sharing Statement

Iwakura. Resignation in Working Women With Breast and Gynecologic Cancers. *JAMA Netw Open*. Published August 25, 2025. doi:10.1001/jamanetworkopen.2025.28844

**Data**
**Data available:** No

**Additional Information**
**Explanation for why data not available:** The data supporting the findings of the current study are available from the Japan Health Insurance Association. However, we used this data under license for this study, so it is not publicly accessible. Supplement 2 provides this context.
